# Evidence based clinical practice guideline for follow-up care in persons with spinal cord injury

**DOI:** 10.3389/fresc.2024.1371556

**Published:** 2024-09-09

**Authors:** Inge Eriks-Hoogland, Lorena Müller, Michael Baumberger, Burkhart Huber, Franz Michel, Celina Belfrage, Hicham Elmerghini, Mide Veseli-Abazi, Ralf Böthig, Kai Fiebag, Roland Thietje, Xavier Jordan

**Affiliations:** ^1^ Department of Paraplegiology, Swiss Paraplegic Centre, Nottwil, Switzerland; ^2^ Faculty of Health Sciences and Medicine, University of Lucerne, Lucerne, Switzerland; ^3^ Department of Health Services and Clinical Care, Swiss Paraplegic Research, Nottwil, Switzerland; ^4^ Department of Traumatology, AUVA Rehabilitation Centre, Häring, Austria, Switzerland; ^5^ Department of Paraplegiology, REHAB Basel, Basel, Switzerland; ^6^ Centre for Spinal Injuries, BG Trauma Hospital Hamburg, Hamburg, Germany; ^7^ Department of Paraplegiology, Clinique Romande de Réadaptation, Sion, Switzerland

**Keywords:** spinal cord injury, spinal cord disease, spina bifida, lifelong follow-up care, prevention, outpatient care, guideline

## Abstract

**Introduction:**

While it is well-established that follow-up care programs play a crucial role in preventing and early detecting secondary health conditions (SHCs) in persons with spinal cord injury [SCI, including spina bifida (SB)], the availability of evidence-based follow-up care programs remains limited. Under the leadership of the German-speaking Medical Society for Paraplegiology (DMGP), we have developed an evidence based clinical practice guideline for follow-up care of SHCs in persons with SCI and identify research gaps.

**Methods:**

This guideline was developed in accordance with the regulations of the Association of the Scientific Medical Societies in Germany (AWMF e.V.). To ensure an evidence-based guidance, we utilized the International Classification of Functioning, Disability and Health (ICF) generic core set and ICF Core Set for individuals with SCI in long-term context as our foundational framework. We conducted a comprehensive literature review to identify existing recommendations for follow-up care and graded the level of evidence according to relevant instruments. Subsequently, we formulated recommendations and achieved consensus through a structured nominal group process involving defined steps and neutral moderation, while adhering to the criteria outlined in the German guideline development instrument (DELBI).

**Results:**

Although there is a fair number of literatures describing prevalence and severity of SHCs after SCI, the amount of literature including recommendations was low (19 for SCI and 6 for SB). Based on the current evidence on prevalence and severity of SHCs and available recommendations, a clinical practice guideline on follow-up care of most relevant SHCs was defined. The recommendations for follow-up care are described in the following chapters: (1) Nervous system; (2) (Neuropathic) pain; (3) Cardiovascular diseases; (4) Respiratory System; (5) Immunological system, vaccination and allergies; (6) Gastrointestinal tract and function; (7) Endocrinological system and nutrition; (8) Urogenital system; (9) Contraception, pregnancy, birth and postpartum care; (10) Musculoskeletal system; (11) Pressure injuries; (12) Psychological health; (13) Medication and polypharmacy.

**Conclusion:**

We could successfully establish an evidence based clinical practice guideline for follow-up care of SHCs in individuals with SCI. There is however a notable lack of high-quality recommendations for SCI follow-up care.

## 1 Introduction

Spinal cord injury (SCI) is a complex and debilitating medical condition that often results in profound and long-lasting physical and neurological impairments, leading to functional, inclusional, psychological, and socioeconomic challenges. On a population level, advances treatment and in medical rehabilitation techniques have increased the lifespan of individuals with SCI (1–3), but life expectancy is still lower than the general population (4, 5). There is a substantial variation in mortality and longevity within the SCI population, between WHO regions, and country income levels (6). The reasons for this variation are complex and may include factors such as income, access to healthcare, and social support. On an individual level, the traditional view of SCI as a relatively stable condition has given way to a more nuanced perspective that recognizes aging with SCI as a multidimensional and complex process encompassing physical, psychological, and social changes (7). The management of SCI therefore goes beyond the initial injury and acute care phase, necessitating a comprehensive approach to long-term follow-up care. A critical aspect of follow-up care involves the prevention or early diagnosis of new health conditions (HCs).

HCs following SCI are not only common but also significant determinants of disability, reduced life satisfaction, emotional well-being (8), as well as leading cause for increased mortality and diminished life expectancy (9). These HCs encompass a wide range of physical and psychological diagnoses and symptoms stemming from impairments, activity limitations, and participation restrictions, and are often referred to as “secondary” HCs (SHCs). Common and recurrent SHCs include chronic nociceptive and neuropathic pain, spasticity, urinary tract and pulmonary infections, circulatory problems, osteoporosis and related fractures, bowel and bladder regulation issues, sexual dysfunction, and pressure injuries. Furthermore, individuals living with SCI may face an elevated risk of chronic diseases associated with the general aging process, such as diabetes, cardiovascular disease, breast and cervix cancer, and bladder cancer (10), potentially contributing to the observed difference in survival rates between persons with SCI and the general population. Leading causes for pre-mature death in SCI are respiratory complications and heart diseases (1, 2, 4, 11–22). A challenging group in follow-up care are persons with Spina Bifida (SB). Despite advancements, such as implantation of shunts and regular urological check-ups, individuals with SB continue to face excess morbidity and mortality into adulthood (12–15). Depending on the severity of neurological impairment, only 17%–61% of affected individuals survive to the age of 40 (16).

Despite the established benefits of follow-up care programs throughout the lifespan, including improved health, prevention of SHCs, current follow-up care programs are scarce, limited in availability, predominantly rely on expert opinions, and exhibit considerable variation in terms of content, frequency, and setting (23). To provide individuals with SCI with up-to-date and optimal medical and rehabilitative care, as well as to establish a first step building an evidence-based framework for a (learning) health system (LHS) in SCI, the development of an evidence-based clinical practice guideline for follow-up care in persons with SCI is imperative.

The German-speaking Medical Society for Paraplegiology (DMGP) has, therefore, tasked its members with the development of a clinical practice guideline for the follow-up care of SHCs in individuals with SCI. This guideline aims to provide the most comprehensive, evidence-based, and current recommendations for the long-term care of persons with SCI. Its purpose is to serve as a foundation for a dynamic LHS, ensuring continuous enhancement through structured data assessment along the continuum of care and so building a framework for research and adaptation. The guideline addresses critical clinical questions, including:

1. *Content of follow-up care programs:* What should be the core components of follow-up care programs focusing on prevention and early adaptation of SHCs?
2. *Assessment*: Which assessments, including clinical and additional evaluations, should be conducted?
3. *Assessments tailored to specific SCI groups*: Are there specific assessments that should be uniquely tailored to distinct SCI groups, such as different age groups, individuals with tetraplegia, those with complete lesions, persons able to walk, women, and persons with SB?
4. *Frequency of follow-up care appointments:* How often should individuals undergo follow-up care appointments?
5. *Setting for follow-up care appointments*: In what clinical settings should these follow-up care appointments take place?

By addressing these clinically relevant key questions, the guideline seeks to provide a structured and evidence-based approach to optimize the long-term care of individuals with SCI, ultimately aiming to enhance their overall well-being and quality of life.

## 2 Methods

### 2.1 Members of the guideline development group

The development of the present guideline was spearheaded by the DMGP e.V. The core working group comprised six medical doctors/physicians (IEH, MB, RT, BH, FM, XJ) all with over 15 years of experience in the field of SCI, and a clinical scientist (LM). The extended working group existed of medical doctors/physicians hailing from diverse backgrounds including physical medicine and rehabilitation, internal medicine, neurology, gynecology, traumatology, SB, neurorehabilitation, pneumology, and neuro-urology (KF, HE, CB, MVA, RB) as well as members from patient associations for individuals living with SCI or SB. The development process also engaged representatives (mandates holders) of professional societies within the Association of the Scientific Medical Societies (AWMF) for external reviews. Prior to submission to the AWMF, all DMGP members were provided the opportunity to review the guideline.

### 2.2 Guideline development process

**Figure 1:**
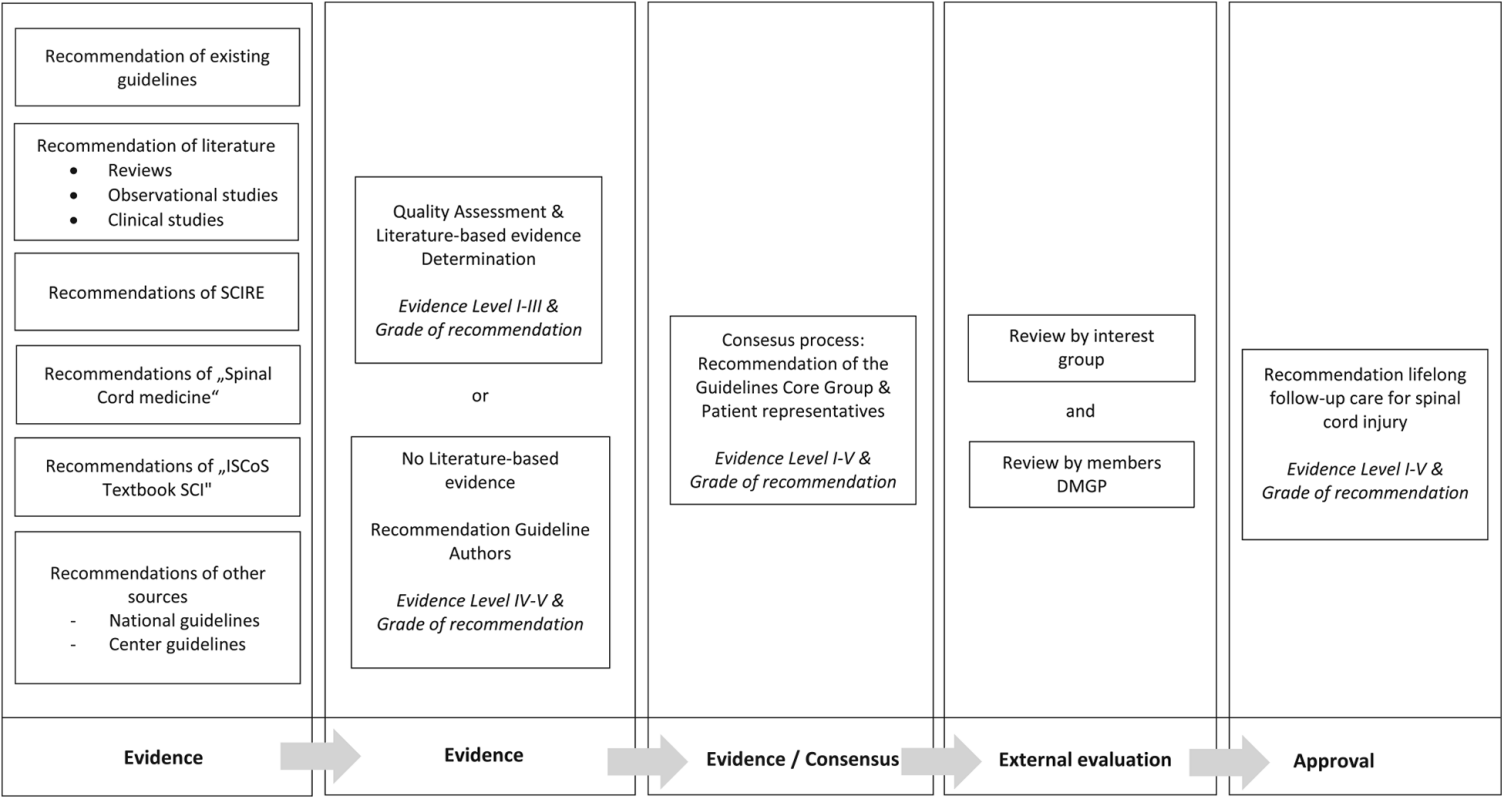
Guideline development process.

The development process adhered to the criteria outlined by the AWMF, ensuring that the resulting guideline is not merely an expert opinion but an evidence and consensus-based guideline (see awmf.org). The core group initiated the guideline development process by identifying pertinent areas of focus, using the ICF core set (24) and ICF core sets for SCI (25) as a framework and iteratively crafting draft recommendations. Subsequently, the developed recommendations were subject to in-depth discussions during face-to-face and virtual meetings. Over the course of eight guideline committee meetings, consensus on final recommendations was achieved through a structured nominal group process facilitated by neutral moderation. The draft guideline was then disseminated to the extended group for review. In a subsequent step, the representatives (mandates holders) of the professional societies within the AWMF were involved in the guideline for voting within their respective boards, serving as an external review process. During this phase, these representatives assessed and provided comments on the draft guideline. As part of the consensus-building process and prior to submission and publication of the consented guideline to the AWMF, an external review was conducted by all DMGP members, who were given 4 weeks to offer feedback on the guideline (Figure 1).

### 2.3 Content of the guideline

The content of the clinical practice guideline was structured using the International Classification of Functioning and Health (ICF) Generic Core Set and ICF Core Sets for individuals with spinal cord injury in the long-term context as frameworks (Table 1) (26, 27). Additional ICF categories were incorporated by expert opinion recommendation of the core group (see Supplementary Material).

**Table 1:** ICF categories of the guideline.

Second level	Third level	Fourth level	Title	Categories in ICF core set	Additional categories added by the core group	Decision of the core group	Relevant health condition	Chapter
b126			Temperament and personality functions	✓		Inclusion	Adjustment disorder, depressionen & suicide	Psychological disorder
b130			Energy and drive functions	✓		Inclusion	Adjustment disorder, depressionen & suicide	Psychological disorder
b134			Sleep functions	✓		Inclusion	Sleeping problems	Nervous system
b152			Emotional functions	✓		Inclusion	Adjustment disorder, depressionen & suicide	Psychological disorder
b164			Higher level cognitive functions		✓	Inclusion	Impairment of cognitive function (including SHT, TP, spina bifida)	Nervous system
b260			Proprioceptive function	✓		Inclusion	Function of the nervous system in relation with function of the upper extremities	Nervous system, musculoskeletal system
b265			Touch function	✓		Inclusion	Loss of sensibility	Nervous system, musculoskeletal system, skin
b270			Sensory functions related to temperature	✓		Inclusion	Loss of perception of temperature and other stimuli	Skin
		b28010	Pain in head and neck	✓		Inclusion	Pain HWS, neuropathic pain	Neuropahtic pain, musculoskeletal system
		b28011	Pain in chest	✓		Inclusion	Cardiovascular problems, neuropathic pain	Cardiovascular system, neuropathic pain
		b28012	Pain in stomach or abdomen	✓		Inclusion	Reflux, magacolon, obstipation, analfissuren, hemorrhoids	Digestive system and neurogenic bowel dysfunction
		b28013	Pain in back	✓		Inclusion	Scoliosis, neuropathic pain	Musculoskeletal system, neuropathic pain
		b28014	Pain in upper limb	✓		Inclusion	Pain upper extremities, neuropathic pain	Musculoskeletal system, neuropathic pain
		b28015	Pain in lower limb	✓		Inclusion	Pain lower extremities, neuropathic pain	Musculoskeletal system, neuropathic pain
		b28016	Pain in joints	✓		Inclusion	Pain lower and upper extremities	Musculoskeletal system
	b2803		Radiating pain in a dermatome	✓		Inclusion	Neuropathic pain, tethered cord, adhesions, syrinx	Neropathic pain, nervous system
	b2804		Radiating pain in a segment or region	✓		Inclusion	Neuropathic pain, tethered cord, adhesions, syrinx	Neuropathic pain, nervous sytem
b420			Blood pressure functions	✓		Inclusion	Arterial hypertension, arterial hypotension, autonomic dysregulation	Cardiovascular system, neurvous system
	b4303		Blood clotting functions		✓	Inclusion	Thrombosis	Cardiovasular system
b435			Immunsystem functions		✓	Inclusion	Functions of the immune system, vaccination, allergies	Immune system
b440			Respiration functions	✓		Inclusion	Respiratory disorders & ventilation, respiratory infections, sleep-related breathing disorders	Respiratory system
b445			Respiratory muscle functions	✓		Inclusion	Breathing disorder & ventilation, respiratory infections	Respiratory system
b455			Exercise tolerance functions	✓		Inclusion	Reduction in endurance performance functions, reduction in aerobic capacity, reduction in resilience, reduction in fatigue resistance	Cardiovascular system and respiratory system
b510			Ingestion functions		✓	Inclusion	Dysphagie, reflux (regurgitation)	Digestive, metabolic and endocrine systems
b525			Defecation functions	✓		Inclusion	Dysfunction involving defecation, stool consistency, stool frequency, stool continence, flatulence; dysfunction such as constipation or diarrhea. Megacolon. Annal fissures. Hemmorhoids. Precaution Colonca	Digestive, metabolic and endocrine systems
b530			Weight maintenance functions	✓		Inclusion	Overweight, adipositas, underweight	Cardiovascular, Digestive, metabolic and endocrine systems
b540			General metabolic functions		✓	Inclusion	Diabetes, malnutrition	Digestive, metabolic and endocrine systems
b550			Thermoregulatory functions	✓		Inclusion	Disturbed functions of body temperature maintenance; dysfunctions as in hypothermia, hyperthermia.	Nervous system
b555			Endocrine gland functions		✓	Exclusion	Pubertas praecox, pubertas tarda	Digestive, metabolic and endocrine systems
b610			Urinary excretory functions	✓		Inclusion	Impaired functions of urine collection including risk of renal insufficiency	Urogenital- und reproduktiven System
	b6200		Urination	✓		Inclusion	Impaired functions affecting the emptying of the urinary bladder	Urogenital- und reproduktiven System
	b6201		Frequency of urination	✓		Inclusion	Impaired functions involved in the frequency with which bladder emptying occurs.	Urogenital- und reproduktiven System
	b6202		Urinary continence	✓		Inclusion	Impaired functions involved in the control of bladder emptying	Urogenital- und reproduktiven System
b640			Sexual functions	✓		Inclusion	Disturbed sexual function	Urogenital- und reproduktiven System
b660			Procreation functions	✓		Inclusion	Reduced fertility, anticonceptives, pregnancy, birth	Urogenital- und reproduktiven System
b670			Sensations associated with genital and reproductive functions	✓		Inclusion	Problems with menstruation (e.g., spasticity, autonomic dysregulation), dysmenorrhea. Disturbance of sensations during sexual intercourse	Urogenital- und reproduktiven System
b710			Mobility of joint functions	✓		Inclusion	Reduced joint mobility	Musculoskeletal system
b715			Stability of joint functions	✓		Inclusion	Reduced stability of the joints, e.g., shoulder	Musculoskeletal system
b720			Mobility of bone functions	✓		Inclusion	Reduced mobility of bones, i.e., scapula and pelvis	Musculoskeletal system and skin
b730			Muscle power functions	✓		Inclusion	Reduced muscle strength function	Nervous system
b735			Muscle tone functions	✓		Inclusion	Altered muscle tone, e.g., spasticity	Nervous system
b740			Muscle endurance functions	✓		Inclusion	Reduced muscle strength & endurance function	Musculoskeletal system
b750			Motor reflex functions	✓		Inclusion	Disruption of involuntary muscle contractions	Nervous system
b760			Control of voluntary movement functions	✓		Inclusion	Disturbance of control and coordination due to, for example, spasticity and loss of muscle strength.	Nervous system
b770			Gait pattern functions	✓		Inclusion	Disturbance of the functions of the movement pattern during walking	Nervous system, musculoskeletal system
b780			Sensations related to muscles and movement functions	✓		Inclusion	Altered muscle tone, e.g., spasticity	Nervous system
b810			Protective functions of the skin	✓		Inclusion	Pressure injury	Skin
b820			Repair functions of the skin	✓		Inclusion	Preussure injury	Skin
b830			Other functions of the skin	✓		Inclusion	Disturbed function of sweating	Nervous system
b840			Sensation related to the skin	✓		Inclusion	Disturbed sensation of the skin (sensitivity)	Nervous system, skin
s110			Stucture of brain		✓	Inclusion	Shunt dysfunction	Nervous system
		s12000	Cervical spinal cord	✓		Inclusion	Syrinx, adhesions, tethered cord	Nervous system
		s12001	Thoracic spinal cord	✓		Inclusion	Syrinx, adhesions, tethered cord	Nervous system
		s12002	Lumbosacral spinal cord	✓		Inclusion	Syrinx, adhesions, tethered cord	Nervous system
		s12003	Cauda equina	✓		Inclusion	Tethered cord	Nervous system
	s1201		Spinal nerves	✓		Exclusion	Disturbance of the function of the spinal nerves	Nervous system
s430			Structure of respiratory system	✓		Inclusion	Tracheostoma	Respiratory system
s589			Structures related to the digestive, metabolic and endocrine systems, other specified	✓		Inclusion	Osteoporose	Digestive, metabolic and endocrine systems
s610			Structure of urinary system	✓		Inclusion	Bladder tumors, other bladder structure changes	Urogenital system
s720			Structure of shoulder region	✓		Inclusion	Changes such as osteoarthritis, rotator cuff	Musculoskeletal system
	s7300		Structure of upper arm	✓		Inclusion	N. Ulnaris Neuropatie	Musculoskeletal system
	s7301		Structure of forearm	✓		Inclusion	Carpal tunnel syndrome	Musculoskeletal system
	s7302		Structure of hand	✓		Inclusion	Arthrosis	Musculoskeletal system
	s7408		Stucture of pelvic region		✓	Inclusion	Gynecological prevention	Urogenital and reproductive system
	s7500		Structure of thigh	✓		Inclusion	Changes e.g., arthrosis, hip dyslplasia	Musculoskeletal system
	s7501		Structure of lower leg	✓		Inclusion	Changes e.g., arthrosis, foot deformities	Musculoskeletal system
s760			Stucture of trunk		✓	Inclusion	Scoliosis	Musculoskeletal system
n.s.					✓	Inclusion	Polypharmacie	Medication

Subsequently, a systematic review was conducted to identify existing evidence and recommendations regarding follow-up care in SCI from January 2010 to December 2018 in MEDLINE. A systematic literature search was executed in PubMed, Cochrane Library, and additional databases for guidelines on the topic of follow-up care. Supplementary searches were conducted in textbooks (see Figure 1) (28, 29) and on the websites of prominent SCI clinics and spina bifida associations (American Spina Bifida Association). Grey literature sources were also examined from the Spinal cord injury research evidence (SCIRE) project and existing guidelines.

The Population, Intervention, Comparison and Outcomes (PICO) tool was used to identify all relevant literature. Included were all studies which included persons with SCI and SB (P), focusing on content, frequency and setting of follow-up care of SHC in SCI (I) and describing outcome measures (O).

A reviewer (IEH) evaluated the identified “titles and abstracts” according to the inclusion criteria (IEH). After an initial selection of literature from PubMed (spinal cord injury *n* = 1,973/SB *n* = 19), the full texts from the databases were then retrieved (*n* = 34/*n* = 6) and evaluated by two reviewers (IEH & LM). After analyzing the full text, the reviewers then decided again whether the study met the established criteria. In case of disagreement, two additional members of the guideline group were consulted to reach a decision. In total, 19/6 articles were ultimately assessed as suitable for addressing the key questions and thus serve as the basis of evidence. The information from the relevant literature was extracted by one person (IEH) and documented in a specially developed template, which was later used for processing in the consensus process. The information includes title and authors of the study, journal, type of study, method, study objective, outcome, result, study quality, and level of evidence. Since all identified texts were written in English, the summary of the information was also conducted in English. Additional findings from guidelines and textbooks were consecutively included in the template.

### 2.4 Determination of level of evidence and grade of recommendation

The approach to evidence assessment is based on the process of a systematic literature review. Critical Appraisal Tools are used to evaluate the quality of the relevant literature. Since various types of literature and studies were identified through the literature search, different tools were also utilized to represent the quality. Level of evidence was classified according to the AWMF Regulations in four Grade categories: high, moderate, low and very low (30).

In a next step the Grade of recommendation A (must/must not), B (should/should not) and 0 (could/could not) was then defined, based on the level of evidence and according to the regulations of the AWMF (30).

**Figure 2:**
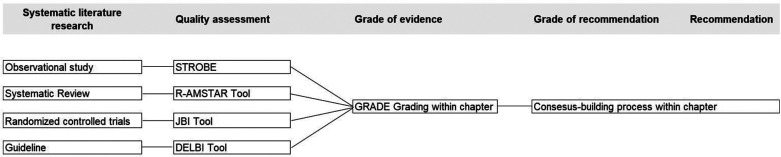
Process systematic literature review, quality assessment, grading of evidence and grade of recommendation.

A detailed description of the systematic review (including all used sources, used search term per database) is available in German on the AWMF-Website (31) and is submitted as a separate manuscript for publication. Details on the review-process can be requested by emailing the correspondent author. In a next step, quality assessments were performed in accordance with AWMF regulations for all guidelines and peer-reviewed literature. Figure 2 illustrates the process of quality assessment, determination of evidence levels, and formulation of recommendations. The analysis of this literature search served as the foundation for this guideline.

## 3 Results

### 3.1 Content of the guideline

Based on the ICF Generic Core Set (27) and ICF Core Sets for individuals with spinal cord injury in the long-term context (26) and expert opinion of the working groups a content of the guideline was defined (see Supplementary Table S1). The included ICF categories were clustered in 13 chapters: (1) Nervous system, including syringomyelia, tethered cord, adhesions of the spinal cord, cognitive function, shunt function, spasticity, sleep and autonomic function; (2) Pain; (3) Cardiovascular diseases including cardiometabolic syndrome and thrombosis; (4) Respiratory System, including breathing function, ventilatory assistance, infections and sleep-associated breathing disorders; (5) Immunological system including vaccination and allergies; (6) Gastrointestinal tract and function including obstipation and incontinence, megacolon, anal fissure and anal fistula, hemorrhoids, screening recommendations for colon carcinoma and dysphagia; (7) Endocrinological system and nutrition including osteoporosis and malnutrition; (8) Urogenital system, including bladder function, renal function and urinary tract infections, preventive medicine and screening for women, menstruation, and contraception, sexual function and fertility in men, and sexuality, fertility, and pregnancy in women with spinal bifida; (9) Pregnancy, birth and postpartum care; (10) Musculoskeletal system including upper extremities, lower extremities, and scoliosis; (11) Pressure injuries; (12) Psychological health; (13) Medication and polypharmacy.

All information of the included studies, guidelines and textbooks was allocated to the referring chapters and authors summarized the relevance and recommendations for follow-up care.

### 3.2 Recommendations for follow-up care appointments

The AWMF-guideline “Follow-up care in persons with SCI” (32) recommends following: The primary objectives of these appointments are to prevent the onset of SHCs, facilitate early diagnosis of HCs, and optimize the person's rehabilitation status.

1. Content of follow-up care: Follow-up care appointments should encompass a broad range of health aspects, as defined by the current guideline (see Supplementary Table S1) and not be limited to SCI-specific issues. They should also evaluate typical aging-related concerns, such as degenerative changes and cardiovascular health, which can be exacerbated by SCI.
2. Assessment: Ideally, follow-up care appointments should adopt an interdisciplinary and/or interprofessional approach, involving healthcare professionals from various backgrounds. This collaborative approach ensures comprehensive assessment and management of the person's health. In Supplementary Table S1 specific recommendations on assessments, including clinical and additional evaluations, are made.
3. Frequency of follow-up care appointments: Regular monitoring for all individuals with SCI: It is strongly recommended that every person with SCI undergoes regular follow-up care appointments. It is advisable to schedule follow-up appointments at specific intervals. Initially, after primary rehabilitation, persons should attend follow-up appointments at 3, 6, and 12 months. Subsequently, annual appointments at specialized SCI clinics are recommended.
4. Setting of follow-up appointments: Follow-up care appointments ideally are scheduled at a specific SCI clinic to ensure a holistic health evaluation. Specific health issues might need inclusion of other medical specialists, an important role in healthcare management of persons with SCI is reserved for the general practitioner of the person.
5. Assessments tailored to specific SCI groups: Specific recommendations for different age groups, persons with tetraplegia, those with complete lesions, persons able to walk, women, and persons with spina bifida are made where applicable and included in Supplementary Table S1.

### 3.3 Key components of follow-up care appointments

The supplement provides a detailed overview of recommendation of all above mentioned 13 chapters, but now clustered in five key-components (neurological status, general internal status, neuro-urological & urogenital status, musculoskeletal status and rehabilitation status). For each chapter a short description of the problem (frequency/severity) is given, followed by the recommendations including level of evidence and grade of recommendation. In Supplementary Table S1, we provide a “quick-read” overview of the content of follow-up care appointments, which include medical history, as well as clinical examination and additional examinations, if needed. It also includes an overview of assessments that are recommended.

The key components of follow-up care appointments are listed below. They mirror the comprehensive approach which is needed to ensure that all relevant aspects of health and functioning are covered in follow-up appointments.

1. SCI specific neurological assessment: Each follow-up care appointment should include a thorough assessment of SCI-specific neurological aspects (33). This evaluation should encompass the individual's neurological status, including motor and sensory function, as well as any changes or developments in their SCI. In persons with SB, special attention should be given to cognitive status and shunt function. Any neurological concerns or developments should be identified and addressed.
2. Assessment of internal medicine status: The follow-up care process should involve a comprehensive assessment of the individual's internal medicine status. This includes evaluating their overall health (including for example cardiac, respiratory and bowel status), but also mental health status, vaccination status, preventive care and screening status and medication management. Any changes or issues related to internal medicine should be addressed and managed as appropriate.
3. (Neuro-) musculoskeletal assessment: An integral part of follow-up care is the assessment of (neuro-) musculoskeletal health. This evaluation examines the individual's musculoskeletal system, including bone health, joint function (including spine), and muscle strength. Any musculoskeletal concerns or developments should be identified and addressed.
4. Neuro-urological and urogenital assessment: Follow-up care appointments should incorporate a neuro-urological and urogenital assessment. This includes evaluating urinary and genital function, identifying any issues related to bladder or sexual health, and addressing them as needed.
5. Rehabilitation status: The individual's rehabilitation status should be continuously monitored and assessed during follow-up care appointments. This involves evaluating progress, rehabilitation goals, and the need for adjustments or modifications to the rehabilitation plan.

By adhering to these recommendations, individuals with SCI can receive comprehensive and proactive care that aims to optimize their overall well-being, manage SCI-specific challenges, and address potential health issues associated with aging. This approach ensures that follow-up care is tailored to the unique needs of each person with SCI, promoting a higher quality of life and long-term health.

## 4 Discussion

The current clinical practice guideline for follow-up care in individuals with SCI represents a significant milestone as it stands as the first comprehensive, evidence-based guideline for follow-up care across the lifespan for individuals with SCI and SB. This guideline emerged through a collaborative effort of experts, including healthcare specialists and individuals with lived experience. The incorporation of the ICF generic core set and the ICF core set for SCI underscores the guideline's robust evidence-based foundation, elevating it beyond mere expert opinion. With the implementation of the current follow-up care guideline, a framework for assessment of SHCs in persons with SCI has now been established, making outcomes of follow-up carte appointments measurable and therefore comparable, within and between health settings. It also sets the standard for follow-up care recommending on content, frequency of follow-up care appointments and specific considerations for subgroups, such as persons with tetraplegia and SB.

However, it is important to acknowledge a notable challenge faced during the guideline's development, given the scarcity of evidence and quality (no randomized controlled trials) and the limited quality of evidence concerning the frequency of follow-up care. This limitation necessitated reliance on indirect evidence, considering factors such as the severity of SHCs, the frequency of occurrence, their modifiability, and expert opinions. For example, a significant research gap was found for prevalence of breast and cervical cancer in women with SCI and adherence to preventive care programs, recommendations on vaccinations for persons with SCI, or specific recommendations for the ageing population. The existence of this research gap underscores the need for further investigation, in this area to enhance the evidence base for future guideline updates. Implementation of defined assessments in clinical practice, making routine clinical data available for research will be a next step in improving quality of care. The evidence-based clinical practice guideline for follow-up care in SCI represents a significant advancement in evidence-based care, serving as a comprehensive resource for individuals with SCI and SB across their lifespan. In addition to its clinical applications, the guideline acknowledges the potential utility of clinical data for research purposes (27). In its nature, it is part of an ongoing endeavour to improve the clinical care of persons with SCI and SB using a LHS approach (28). The idea of a LHS assumes that a health system can learn when it can rely on cyclic processes where reliable and regular assessed data for the health system serve as a basis for the generation of new evidence. The evidence is transferred into practice for implementation, and structured data generated from practice as well as experience from implementation are fed back into the cycle. By including structured assessments and outcomes, a comparison over time and between health care settings is possible. In recognition of the dynamic nature of SCI and the ever-evolving healthcare landscape, the guideline adopts a learning health system approach. This approach serves as a bridge between research evidence and practical application, allowing real-world data to generate new research questions and inform ongoing improvements in care delivery. Consequently, the guideline remains adaptable and aligned with the dynamic nature of SCI and healthcare.

It is imperative to emphasize that guidelines are most effective when integrated into everyday clinical practice. The implementation of the guidelines in our health care setting is a step-wise approach based on recommendations of Beachemin et al. (34) using a repeating-process model. While the guideline provides a valuable framework for follow-up clinical care, it is essential to underscore that individualized care should always consider the unique circumstances of each health care setting and each person. Guidelines are not legally binding documents and should be applied judiciously in light of individual needs, and the regional/national health care system.

The guideline is anticipated to undergo periodic updates in accordance with AWMF guidelines. These updates will prioritize the enhancement of guideline development methodology, including the evaluation of systematic reviews and meta-analyses in line with the Preferred Reporting Items for Systematic Reviews and Meta-analysis (PRISMA) statement and the application of the GRADE approach to assess the quality of available evidence and the strength of recommendations.

Finally, follow-up care of persons with SCI encompasses much more as prevention and early treatment of SHCs. Besides the assessment and evaluation of body structures and body functions, regular evaluation and assessment of activities, participation, environmental factors, personal factors, and quality of life should be performed during each follow-up visit. The ICF and it's core sets (24, 25) build a framework for the evaluation of all relevant aspects of functioning with SCI. The current guideline show already that various health care specialists are involved in the prevention and early diagnosis of SHCs (among which, Physical Medicine and Rehabilitation, neurologist, neuro-urologist, Physiotherapy, Occupational Therapy etc.) and coordination of care is complex. From earlier studies we know that persons with SCI frequently contact their general practitioner for SCI related health care issues (35, 36). The guideline recommends specialist in SCI care (ideally a PMR specialist) to be the coordinator of follow-up care, in close collaboration with all included health care specialist, including the general practitioner. The guideline therefore is not only a tool for SCI specialists, but also informs general practitioners and persons with SCI on the recommendations for follow-up care.

## 5 Conclusion

The DMGP successfully established a clinical practice guideline for follow-up care of SHCs in individuals with SCI. Based on the current evidence on prevalence and severity of SHCs and available recommendations, a clinical practice guideline on follow-up care of most relevant SHCs was defined.

The recommendations for follow-up care are described in the following chapters: (1) Nervous system; (2) (Neuropathic) pain; (3) Cardiovascular diseases; (4) Respiratory System; (5) Immunological system, vaccination and allergies; (6) Gastrointestinal tract and function; (7) Endocrinological system and nutrition; (8) Urogenital system; (9) Contraception, pregnancy, birth and postpartum care; (10) Musculoskeletal system; (11) Pressure injuries; (12) Psychological health; (13) Medication and polypharmacy.

There is however a notable lack of high-quality recommendations for SCI follow-up care. Although we found a fair number of literatures describing prevalence and severity of SHCs after SCI, for some health conditions this was completely missing (for example breast and cervical cancer prevalence in women with SCI and the amount of literature specifying recommendations for follow-up care appointments was low (19 for SCI and 6 for SB) and mostly of low-moderate quality.
