# Supplementary material for: Evidence based clinical practice guideline for follow-up care in persons with spinal cord injury
Source: Front Rehabil Sci. 2024 Sep 9;5:1371556. doi: 10.3389/fresc.2024.1371556 (PMC11417090; doi:10.3389/fresc.2024.1371556)
Supplement: Supplementary file 1 [file Table1.docx]

Supplementary Material

# Supplementary Data

(LoE= Level of evidence, GoR= Grade of recomendation)

**Chapter 1: Nervous system**

A regular neurological evaluation is recommended for all individuals with spinal cord injury (SCI) (e.g. International Standards for Neurological Classification of Spinal Cord Injury (S. C. Kirshblum et al., 2011). This evaluation is crucial, not only during the initial rehabilitation, but also as an ongoing practice to tailor and optimize outpatient rehabilitation programs according to the current neurological status. Additionally, regular assessments are essential to facilitate the early diagnosis of significant Secondary Health Conditions (SHCs), such as syringomyelia, tethered cord syndrome (TCS), and carpal tunnel syndrome (CTS).

***1.1 Syringomyelia, Tethered Cord Syndrome, and adhesions of the spinal cord (ICF: s12000-12003)***

While the prevalence of syringomyelia development may be relatively low (1-7%), its consequences can be disastrous (Carroll & Brackenridge, 2005; el Masry & Biyani, 1996; Krebs, Koch, Hartmann, & Frotzler, 2016; Schurch, Wichmann, & Rossier, 1996). In the development of an intraspinal cavity formation, there is often a deterioration in neurological status. Other typical symptoms include increasing or newly occurring pain, an increase in spasticity, and a change in neurological levels. The course is typically gradual and, in part, progressive and variable (Ali, Preci Hamilton, & Yakoub, 2018; Carroll & Brackenridge, 2005). The literature indicates that early surgery yields better outcomes (Bonfield, Levi, Arnold, & Okonkwo, 2010; Klekamp, 2012; Silberstein & Hennessy, 1992). LoE: high

Tethered cord syndrome (TCS) and spinal cord adhesions are conditions typically associated with spina bifida but may also manifest in individuals with SCI, particularly those who have undergone surgery, experienced spinal bleeding, or had infections (Lew & Kothbauer, 2007). Due to these facts, regular neurological assessment and possibly the detection of syringomyelia are necessary. LoE: high

Recommendations:

- During each follow-up appointment, healthcare providers must inquire about changes in neurological status, especially deterioration and any symptoms that could suggest the development of syringomyelia, TCS, or spinal cord adhesions. GoR: A
- If the individual reports neurological deterioration or new symptoms, a neurological assessment must be conducted. GoR: A
- In case of a clear deterioration of neurological symptoms and suspicion of syrinx formation, even years after the trauma, an MRI must be promptly performed. The cranial and caudal ends of the syringomyelia should always be visualized. High-resolution CISS ("constructive interference in steady state") sequences are helpful in depicting arachnoidal adhesions. In addition to CISS images, pulse-triggered phase-contrast cine-mode images in sagittal projection should be obtained for the direct visualization of pulsation obstruction (Mauer, Freude, Danz, & Kunz, 2008). GoR: A
- Upon confirmation of syringomyelia or TCS, a neurosurgical evaluation must be initiated to assess the indication for surgery. GoR: A

Level of agreement: 100%***1.2 Cognitive function (ICF b164)***

Cognitive function may be compromised in individuals with SCI, particularly those with tetraplegia and those who have experienced polytrauma (Budd et al., 2017). Changes in cognitive function are more frequently observed in individuals with spina bifida, especially those with hydrocephalus (Bowman, McLone, Grant, Tomita, & Ito, 2001). LoE: high

Recommendations:

- As part of lifelong follow-up care, individuals diagnosed with traumatic brain injury (TBI), tetraplegia, or SCIs following high-speed trauma must be actively screened for cognitive problems. GoR: A
- In the context of lifelong follow-up care, individuals with spina bifida (with hydrocephalus/shunt) must be actively screened for cognitive problems, including verbal IQ and performance IQ, visual analysis, and visuomotor function. GoR: A
- If there is a change in higher cognitive function in individuals with spina bifida with a shunt, a review of shunt function must be conducted. GoR: A
- Level of agreement: 100%

***1.3 Spasticity/spastic syndrome (ICF b735)***

Spasticity is a symptom of an upper motor neuron injury. It can cause functional limitations, pain, skeletal deformation, and joint contractures. Adequate diagnosis, clinical examination, and understanding established treatment options can optimize patient care (Billington, Henke, & Gater, 2022). Spasticity is reported in over 70% of individuals with SCI (Brinkhof et al., 2016), and changes (increase, decrease, change in pattern or frequency) may indicate an underlying health condition. A newly occurring spastic movement disorder or altered spastic movement disorder can be indicative of an underlying health problem (e.g., constipation, undiscovered fracture, thrombosis, urinary tract infection, etc.) (Kheder & Nair, 2012). LoE: high

Recommendations:

- During follow-up care appointments, healthcare providers must inquire about any changes in spasticity. GoR: A
- If the individual reports an increase in spasticity, bothersome spasticity, or a noticeable decrease in spasticity, the cause of the change must be investigated. GoR: A
- Level of agreement: 100%

***1.4 Sleep (ICF 134)***

Insomnia is reported by 55-60% of individuals with SCI (Buzzell, Chamberlain, Schubert, et al., 2020; Shafazand, Anderson, & Nash, 2019). People mention restless leg syndrome, difficulties falling asleep, waking up due to pain or spasticity, or periodic limb movement (Shafazand et al., 2019; Telles, Alves, & Chadi, 2011). Insomnia is more prevalent among individuals aged 45-60 years or those experiencing financial problems or pain (Buzzell, Chamberlain, Schubert, et al., 2020). LoE: high

Recommendations:

- As part of follow-up care, individuals with SCI should be asked about any sleep problems they may be experiencing. GoR: B
- For further recommendations, we refer to the AWMF guidelines on "Non-restorative Sleep/Sleep Disorders - Chapter Insomnia in Adults" (Mayer, 2017), "Insomnia in Neurological Disorders" (Mayer, 2020), and "Non-organic Sleep Disorders" (Prehn-Kristensen, 2018).
- Level of agreement: 100%

***1.5 Autonomic dysfunction/Autonomic dysregulation***

Autonomic dysfunction and dysregulation (AD) may be common in individuals with SCI, particularly those with a lesion level above T6 and complete lesion (Karlsson, 1999; Andrei V Krassioukov, Furlan, & Fehlings, 2003; A. V. Krassioukov et al., 2007; Lindan, Joiner, Freehafer, & Hazel, 1980) and can be a potentially life-threatening condition (Bjelakovic, Dimitrijevic, Lukic, & Golubovic, 2014). LoE: high

Recommendations:

- As part of lifelong follow-up care, individuals with a lesion T6 and above must be asked about symptoms of autonomic dysfunction (orthostatic hypotension, cardiac arrhythmias, temperature dysregulation) and autonomic dysreflexia (episodic hypertensive crisis), or newly occurring autonomic dysfunction and dysreflexia. GoR: A
- In the context of lifelong follow-up care, individuals with a lesion above T6 and above should have blood pressure and pulse measured (to determine baseline blood pressure and pulse). GoR: B
- If there are anamnestic symptoms of (newly occurring) autonomic dysfunction, a detailed history must be taken to identify possible causes. GoR: A
- Clinical examination for possible causes must be conducted in individuals with symptoms of AD. GoR: A
- In individuals where no cause for AD could be found through history and clinical examination, additional diagnostics must be initiated (neuro-urology, colonoscopy, radiological examination (spine, fractures, syrinx, tethered cord, adhesions)). GoR: A
- Level of agreement: 100%

**Chapter 2: Pain (ICF b280)**

Pain is a prevalent issue affecting more than 70% of individuals with SCI (Brinkhof et al., 2016; Dijkers, Bryce, & Zanca, 2009; van Gorp, Kessels, Joosten, van Kleef, & Patijn, 2015). It is often linked to conditions such as depression, anxiety, and sleep disturbances (Castro & Daltro, 2009; Gore et al., 2005; Gormsen, Rosenberg, Bach, & Jensen, 2010). Furthermore, pain has a detrimental impact on an individual's participation and quality of life (Dermanovic Dobrota et al., 2014; Gustorff et al., 2008; McDermott, Toelle, Rowbotham, Schaefer, & Dukes, 2006). LoE: high

Recommendations:

- During each follow-up care appointment, healthcare providers must inquire about the presence of pain. GoR: A
- If pain is reported, a focused clinical examination must be conducted. GoR: A
- In case of worsening of pain, determination of the cause is needed (for example due to changes in degree of nerve root/spinal cord compression, tethering, or posttraumatic syringomyelia or there may be the development of a red flag condition.
- Level of agreement: 100%

We direct your attention to the AWMF-Guideline "Pain in SCI" (Steffen et al., 2019).

**Chapter 3: Cardiovascular and metabolic system**

***3.1 Cardiometabolic syndrome (b410-b429, b540)***

The prevalence of metabolic syndrome (Mark S. Nash & Bilzon, 2018; M. S. Nash & Mendez, 2007) and obesity (Gater Jr, 2007) in individuals with SCI is notably higher compared to the able-bodied population, and this risk escalates with age (Banerjea et al., 2008), subsequently leading to increased morbidity and mortality (Cragg, Ravensbergen, Borisoff, & Claydon, 2015). It is now one of the leading causes of mortality in persons with SCI (perhaps due to premature ageing) and cardiovascular evaluation therefore should be integral part of follow-up care(Buzzell, Chamberlain, Eriks-Hoogland, et al., 2020). LoE: high

A clinically useful tool for accurately assessing overweight/obesity in SCI is still lacking (Cragg et al., 2015; Dionyssiotis, 2012; Eriks-Hoogland et al., 2011; Gorgey, Wells, & Austin, 2015). DXA remains the gold standard (Varacallo, 2023). LoE: moderate

Recommendations:

- As part of lifelong follow-up care, regular monitoring of the cardiometabolic syndrome must be conducted in individuals with spinal cord injury (SCI) (to be performed by the primary care physician depending on the healthcare situation). GoR: A
- Since tetraplegics do not exhibit classic symptoms of a heart attack, an annual EKG or long-term EKG should be performed from 10 years after the onset of paralysis or for individuals aged over 60. GoR: B

Specific recommendations:

*Dyslipidemia*

- As part of lifelong follow-up care, the lipid profile should be regularly measured: fasting LDL, TC, TG, and HDL-C. If the test shows no abnormalities, a reassessment should take place every three years. In the presence of multiple risk factors or existing dyslipidemia, annual screening is recommended. GoR: B

*Blood pressure*

- In the context of lifelong follow-up care, blood pressure must be regularly measured (at least annually) in individuals with spinal cord injury. The measurement must always be taken in the same position (sitting or lying). Comparing with previous readings is crucial to identify any changes. GoR: A
- For patients with suspected blood pressure fluctuations, a 24-hour blood pressure *monitoring should be arranged as part of lifelong follow-up care.* GoR: B
- Evaluation of autonomic dysfunction leading to blood pressure abnormalities (hypotension and autonomic dysregulation) should be regularly performed in all patients with level T6 and above. GoR: A

*Obesity*

- As part of lifelong follow-up care, an annual check for obesity/overweight should be conducted. Body weight should be measured annually. Alternatively, BMI can be used with adjusted thresholds (22 kg/m2). GoR: B
- For individuals with suspected or confirmed obesity, close monitoring is recommended. GoR: B
  - In case of a negative test result, reassessment should occur every three years.
  - In case of a positive test result, annual reassessment is advised.

*Diabetes Mellitus*

- As part of lifelong follow-up care, inquire about family history and other risk factors for diabetes mellitus. GoR: B
- Fasting glucose/HbA1c should be measured annually in the context of lifelong follow-up care. GoR: B

*Nutritional Status*

- As part of lifelong follow-up care, an assessment of nutritional status should be conducted for individuals with elevated cardiovascular risk factors. GoR: B
- Level of agreement: 100%

***3.2 Thrombosis (b430)***

The risk of deep vein thrombosis (DVT) and pulmonary embolism (PE) is heightened within the first year following SCI (Azarbal et al., 2011; Kumagai et al., 2020). Pregnancy, hormonal contraceptives, diabetes, smoking, age over 45 years, and complete SCI are associated with an increased risk of thrombosis in SCI (Ghidini, Healey, Andreani, & Simonson, 2008). LoE: high

Recommendations:

- During lifelong follow-up care, specifically in the first year after spinal cord injury (SCI), pregnancy, hormonal contraception, or in the presence of the following risk factors: smoking, diabetes, age >45 years, AIS A (complete spinal cord injury with no motor or sensory function preserved), individuals must be asked about and clinically examined for symptoms of deep vein thrombosis. GoR: A
- Level of agreement: 100%

For further recommendations on thromboembolism prophylaxis, we refer to the AWMF S1 guideline: "Thromboembolism Prophylaxis in Spinal Cord Injury" (Weidner et al., 2020).

**Chapter 4: Breathing, sleep-associated breathing disorders, ventilation, and respiratory infections (b440-b449)**

Respiratory function may be compromised after SCI due to:

Reduction in muscle strength of respiratory muscles, alterations in thoracic and lung compliance, changes in the central nervous system, modifications in respiratory tract anatomy and hyperreactivity or adjustments in the interaction between the thorax and the digestive system (diaphragm) (Berlowitz, Brown, Campbell, & Pierce, 2005; Brown, DiMarco, Hoit, & Garshick, 2006). (LoE: high)

Recommendations:

- As part of lifelong follow-up care, respiratory function, especially in individuals with tetraplegia, must be assessed through spirometry and measurement of inspiratory and expiratory muscle strength. GoR: A
- A symptom-based investigation for sleep-related breathing disorders (recommended due to their frequency and increase with age) and polysomnography should be conducted if there is suspicion. GoR: B
- In the context of lifelong follow-up care, particularly in individuals with higher levels of paralysis, the ability to efficiently clear secretions should be regularly assessed. This involves measuring peak cough flow, maximum inspiratory capacity, and evaluating assisted and mechanically supported cough techniques. GoR: B
- Level of agreement: 100%

For more detailed recommendations, refer to the registered AWMF S2k guideline "Respiration, Respiratory Support, and Ventilation in Acute and Chronic Spinal Cord Injury" (Michel et al., 2022).

**Chapter 5: Immune system, vaccinations, and allergies (b435)**

***5.1 Vaccinations***

The verification of vaccination status and vaccination is, in Switzerland, Germany and Austria, typically carried out by the general practitioner. It is important to note that recommendations for individuals with SCI may vary between countries and may differ from recommendations for the general population.

Influenza: Based on the studies and recommendations from relevant medical societies, all patients with spinal cord injury, regardless of the level of injury, should receive the annual influenza vaccination. German-speaking medical societies (STIKO, BAG, National Vaccination Committee of Austria) also recommend influenza vaccination for high-risk groups. LoE: high

Pneumococcal: Pneumococcal vaccination is consistently recommended for high-risk groups by German-speaking national medical societies (STIKO, BAG, National Vaccination Committee of Austria). The vaccination schedule and preferred vaccine may vary. BAG predominantly sees a benefit for the PCV13 vaccine, while in Austria and Germany, a sequential vaccination with PCV13 and PPV23 is preferred in most cases. The polysaccharide vaccine (PPV23) has been tested for adequate antibody production in individuals with spinal cord injury in the study by Waites, Canupp, Chen, DeVivo, and Nahm (2008). Due to the dynamic nature of vaccination recommendations and the varying availability of vaccines, adherence to the country-specific vaccination schedule with the preferred vaccine is recommended. LoE: moderate-high

Herpes Zoster: Vaccination to prevent herpes zoster and postherpetic neuralgia is recommended by German-speaking medical societies (STIKO, BAG, National Vaccination Committee of Austria) for individuals aged 50 and above, especially for those at risk. LoE: high

Meningococcal: All vaccination recommendations from German-speaking medical societies (STIKO, BAG, National Vaccination Committee of Austria) consider functional or anatomical asplenia as a reason for meningococcal vaccination. The observed spleen atrophy, particularly in lesions above T3 or T1 is a cause of so-called spinal cord injury induced immune deficiency syndrome according to various studies (Brommer et al., 2016). Based on this, we collectively consider the inquiry and discussion of meningococcal vaccination as part of lifelong follow-up care, especially for individuals with spinal cord injury above T3. LoE: moderate

Covid-19: Covid-19 vaccination is recommended by German-speaking national medical societies (STIKO, BAG, National Vaccination Committee of Austria). Due to the dynamic development of vaccination recommendations and the varying availability of vaccines, adherence to the country-specific vaccination schedule with the preferred vaccine is recommended. LoE: moderate

Urinary Tract Infections: Although the clinical benefit is conditionally present for individuals with spinal cord injury, use of Urovaxom® and StroVac® can be discussed as part of lifelong follow-up care. Regarding the prevention of recurrent urinary tract infections, the data for Urovaxom® is more extensive than for StroVac® (Beerepoot, Geerlings, van Haarst, van Charante, & ter Riet, 2013; Pannek, 2011). LoE: moderate

Recommendations:

- As part of lifelong follow-up care, once the basic status is ensured (standard vaccinations, as well as indication and booster shots), specific inquiries about the vaccination status for influenza, pneumococcal, herpes zoster, meningococcal, and Covid-19 must be made, and if necessary, recommendations for these vaccinations must be provided. GoR: A
- For women with recurrent cystitis, vaccination with Urovaxom® can be considered over a period of 3 months. For men, after exhausting other therapeutic options, the use of Urovaxom® can be considered. Intramuscular administration of StroVac® can be considered for both genders. GoR: 0
- Level of agreement: 100%

***5.2 Allergies***

Latex allergy and cross-sensitivity/allergy have been reported in up to 40% of children with spina bifida (Ausili et al., 2007; Spina Bifida Association, 2020), and they have also been observed in individuals with SCI (Monasterio, Barber, Rogers, Able, & Fredrickson, 2000). LoE: moderate.

Recommendations:

- In individuals with spina bifida and SCI, the occurrence of new allergies must be inquired annually. GoR: A
- If new allergies are suspected, a specialist should be consulted. GoR: B.
- Level of agreement: 100%

**Chapter 6:** **Gastrointestinal tract and function (b540)**

SCI has implications for numerous organ systems, including the gastrointestinal tract, primarily leading to fecal incontinence and severe constipation. This issue is collectively termed "neurogenic bowel dysfunction" (nDFS) and significantly impacts the quality of life (Emmanuel, 2010). Individuals with SCI experience bloating, abdominal discomfort, fears of uncontrolled bowel movements or inappropriate gas release, as well as the risk of severe constipation or even intestinal obstruction, which accompanies them throughout their entire lives.

nDFS can be managed through therapy and bowel management but cannot be cured. Therefore, it is crucial to pay specific attention to bowel dysfunction as part of lifelong follow-up care. Aging, comorbidities, and medication therapies can influence neurogenic bowel dysfunction. Hence, regular assessment of bowel dysfunction and the associated bowel management is essential within the framework of lifelong follow-up care, including regular screening and potential adjustments to bowel management.

Poorly managed bowel function can lead to complications such as ileus or intestinal obstruction, significantly affecting the participation in the professional lives of individuals with SCI. Neurogenic bowel dysfunction is thus one of the most frequently cited problematic secondary complications in SCI (Brinkhof et al., 2016).

Assessing nDFS poses a challenge as describing functional disorders in their entirety remains a challenge for both patients and healthcare professionals (Burns et al., 2015). Inquiring about bowel habits, measures taken, and existing secondary complications is a crucial part of the medical history during each check-up, requiring sufficient knowledge of common pathologies. Frequency and quantity of bowel movements are not sufficient parameters, as the expression of disorders in a denervated body can take many unexpected forms. A recent review identified 12 different instruments for the clinical assessment of nDFS, with the Neurogenic Bowel Dysfunction Score (Krogh, Christensen, Sabroe, & Laurberg, 2006; Tate et al., 2020) recommended as a supportive tool.

Additional examinations must be selectively scheduled and interpreted, considering SCI-related peculiarities, in case of uncertainties and as part of preventive examinations.

Although bowel transit time and the size of the colon seem not to change during the first two decades, there are indications that symptoms related to constipation may intensify over time. However, evidence regarding changes in nDFS over time is limited. Therefore, targeted medical history and physical examination must be integrated into lifelong follow-up care.

The most common and relevant complications of nDFS, including constipation, incontinence, megacolon, as well as anal fissures, (peri-)anal fistulas, and hemorrhoids, are described in the following subsections.

It is important to notice that gallstone disease might be more prevalent in persons with SCI (Rotter & Larraín, 2003).

More details to the evaluation and treatment of neurogenic bowel problems are available at: AWMF Leitlinie „Neurogene Darmfunktionsstörung bei Querschnittlähmung“ Entwicklungsstufe: S2k Stand 08-2019 AWMF-Register-Nr.: 179-004 (Geng, Böthing, Kurze, Hildesheim, & Leder Eckhart, 2019).

***6.1 Obstipation, megacolon, and incontinence (b540)***

Obstipation (95%) and incontinence (75%) are highly prevalent in individuals with SCI (Emmanuel, 2010). Chronic obstipation can lead to the development of megacolon (prevalence between 25% and 73%) (Harari & Minaker, 2000; Park, Noh, Kim, & Joo, 2013). Additionally, 30% report chronic abdominal pain (Nielsen, Faaborg, Christensen, Krogh, & Finnerup, 2017). Bowel problems are associated with limitations in participation, and optimal bowel management is a crucial goal of SCI rehabilitation (Geng et al., 2019). LoE: high

Recommendations:

- Diet, fluid intake, and bowel management, including time needed, incontinence, and changes in stool must be assessed annually. GoR: A
- A clinical examination of the abdomen, including the anal sphincter, must be performed during annual check-ups. GoR: A
- The impact of bowel management on participation should be evaluated. GoR: B
- In cases of suspected megacolon, an extended evaluation with colon transit-time (CTT) and X-Abdomen (colon contrast/CT) should be conducted. GoR: B
- Information on bowel management, therapy options, and its evaluation could be provided as a crucial aspect of follow-up care. GoR: 0
- Level of agreement: 100%

***6.2 Anal fissures, anal fistula, and hemorrhoids (s540, b810, b820)***

Although anal problems such as anal fissures, fistulas, and hemorrhoids are common in individuals with SCI, there is limited literature on their prevalence (Previnaire, De Bont, Bordi, Senal, & Mortier, 2018). hemorrhoids often result from inadequate bowel management and obstipation (Delcò & Sonnenberg, 1998; Previnaire et al., 2018). In SCI, pain is rarely a symptom, and individuals often present with symptoms like increased spasticity or AD (Faaborg et al., 2014; Flavin, Ando, & Teraoka, 2009). Complications like abscesses can develop without early detection. LoE: moderate

Recommendations:

- Bowel management must be assessed during annual follow-up appointments, including changes in spasticity, AD, pain, and the presence of blood in stool. GoR: A
- In cases of positive answers, a detailed evaluation and rectal exam must be conducted. GoR: A
- A proctoscopy, an easy and cost-effective method for examining the anal/rectal area, should be considered in cases of positive signs and symptoms. The use of lidocaine is recommended in persons with SCI above T7 to manage AD during the examination. GoR: B
- The risk of autonomic dysreflexia, increased spasticity, limitations in daily activities due to seated mobility, and difficulties in temporarily adjusting stool management (softer stool consistency), as well as delayed wound healing, should be considered when choosing the method and deciding on hospital admission. GoR: B
- Level of agreement: 100%

***6.3 Screening for colon carcinoma (s540)***

While the prevalence of colon cancer in SCI is like the able-bodied population (Han, Kim, Lee, & Lee, 2009), the screening procedure poses challenges. Changes in stool consistency and bowel management should always raise concern and warrant further screening (Doubeni et al., 2018). Screening with occult blood tests is not meaningful, as occult blood may be present due to minor injuries related to bowel management methods, as well as the prevalence of hemorrhoids and anal fissures (Geng et al., 2019). Colonoscopy is a safe and meaningful screening method for individuals with SCI, although it is less frequently performed (Hayman et al., 2013). Preparation for colonoscopy in SCI requires several (up to 3-4) days due to slow "Colon Transit Time" and anal sphincter spasticity (Teng, Song, Svircev, Dominitz, & Burns, 2018). Thrombosis prophylaxis might be considered in individual cases before colonoscopy (Gastroenterologie, 2013; Hayman et al., 2013). LoE: moderate - high

Recommendations:

- In cases of changes in stool consistency and bowel management, a detailed evaluation (Bristol Stool Score, NBD Score) could be conducted. GoR: 0
- The screening for colon carcinoma must follow the recommendations for able-bodied individuals, typically starting above 50 years, and repeating every 5-10 years. GoR: A
- Colonoscopy is the preferred screening method for individuals with SCI, and its preparation may take 3-4 days (be mindful of electrolyte shifts). GoR: A
- In individual cases, particularly in persons with SCI above T7 (risk of AD and limited mobility), inpatient preparation and colonoscopy must be considered. GoR: A
- Level of agreement: 100%

***6.4 Dysphagia (b510)***

Dysphagia is especially observed after cervical column operations (Duchac, 2016). Left unrecognized, dysphagia can lead to aspiration, penetration, dietary problems, and speech difficulties (Hayashi et al., 2020; Miles, Jamieson, Shasha, & Davis, 2019). LoE: high

Recommendations:

- An evaluation of possible dysphagia and aspiration in annual follow-up care should be included. GoR: B
- In cases of suspected dysphagia or aspiration, an additional clinical evaluations and, if indicated, use Videofluoroscopy (VFSS) or Functional Endoscopic Evaluation of Swallowing (FEES) must be performed. GoR: A
- Level of agreement: 100%

See also AWMF Leitlinie „Neurogenic Dysphagia". Entwicklungsstufe: S1. Stand 02-2020. AWMF-Register-Nr.: 030-111 (Dziewas, 2020).

***6.5 Reflux (b515)***

Although there are no reported changes in the prevalence of reflux in individuals with SCI (Singh & Triadafilopoulos, 2000), higher reflux prevalence may be suspected based on risk profiles. LoE: high

Recommendations:

- Gastroesophageal reflux disease must be evaluated during annual follow-up care. GoR: A
- In cases of symptoms, further clinical evaluations and endoscopy must be considered. GoR: A

**Chapter 7: Endocrine and nutritional system**

***7.1 Osteoporosis (b729)***

Osteoporosis is highly prevalent in individuals with SCI, leading to increased prevalence of fractures (Bauman & Cardozo, 2015; Jiang, Dai, & Jiang, 2006). Although some studies have shown positive effects of antiresorptive therapy (Bauman et al., 2015; Bubbear et al., 2011; Goenka, Sethi, Pandey, Joshi, & Jindal, 2018; Oleson, Marino, Formal, Modlesky, & Leiby, 2020; Schnitzer et al., 2016; Shapiro et al., 2007), long-term follow-up studies are still lacking. Many non-pharmacological therapies have been studied, but most have small sample sizes and methodological flaws. Functional electrical stimulation might have a positive effect on bone mass but does not show a sustainable effect (BeDell, Scremin, Perell, & Kunkel, 1996; Clark et al., 2007; Dudley-Javoroski et al., 2016; Giangregorio et al., 2005; Goemaere, Van Laere, De Neve, & Kaufman, 1994; Goktepe, Tugcu, Yilmaz, Alaca, & Gunduz, 2008; Kalke, Frotzer, Moosburger, & Wittgruber, 2018; Soleyman-Jahi et al., 2018). LoE: moderate

Recommendations:

- Lifestyle recommendations also apply to individuals with SCI. GoR: A
- Recommendations for follow-up evaluations (DXA, PQCT) should be discussed individually, taking existing guideline recommendations into account. GoR: B
- Level of agreement: 100%

*For recommendations, we refer to the AWMF S1 guideline "Spinal Cord Injury-Associated Osteoporosis," Registration Number: 179-007 (Kalke et al., 2018).*

***7.2 Malnutrition (b1302, b5105, b530, b540, d560, d5701)***

Malnutrition is highly prevalent (17-60%) in individuals with SCI, and significantly higher than in the able-bodied population (Dionyssiotis, 2012; Wong, Derry, Grimble, & Forbes, 2012; Wong, Derry, Jamous, et al., 2012). Those with tetraplegia, ventilator dependency, inactivity, overweight, and individuals with hydrocephalus are at higher risk of developing malnutrition (Flueck & Perret, 2017; Pellicane, Wysocki, & Schnitzer, 2010; Wong, Derry, Jamous, et al., 2012). Malnutrition is associated with pressure sores, overweight, cardiovascular complications, and increased morbidity (Consortium for Spinal Cord Medicine & Paralyzed Veterans of America, 2014; Shin, Chang, Hwang, & Lee, 2018; Wong, Derry, Jamous, et al., 2012). LoE: high

A balanced diet and supplementation can prevent and treat malnutrition (Flueck & Perret, 2017). The Body Mass Index (BMI) has shown to be an invalid method for assessing adiposity as a risk factor for cardiovascular disease. LoE: high

Recommendations:

- Dietary evaluations during annual appointments, ideally using validated assessments for SCI such as the Spinal Nutritional Screening Tool (SNST) should be conducted. GoR: B
- Body weight should be assessed during annual appointments, with more frequent assessments in cases of malnutrition, overweight, or underweight. GoR: B
- Fat-Free Mass (FFM), which is the gold standard for assessing cardiovascular risk, should be evaluated. BMI is not valid for individuals with SCI. GoR: B
- In all individuals with spinal cord injury (but especially in those with recurrent pressure sores and infections), regular laboratory monitoring should be conducted as part of follow-up care (e.g., hematological, chemical, proteins, vitamin D, vitamin C, zinc, lipid profile, selenium, and vitamin B12). (See also the chapter on cardiometabolic syndrome). GoR: B
- Level of agreement: 100%

**Chapter 8: Urogenital System**

***8.1 Bladder function, urinary tract infections, and renal function (b610-b639)***

Lifelong follow-up is essential to prevent severe urinary tract complications and ensure optimal bladder management and quality of life (Blok et al., 2019). LoE: high

There is no established fixed follow-up schedule (Cameron, Rodriguez, & Schomer, 2012). Therefore, both the time interval and the scope of the examinations for early detection and prevention of complications must be defined on an individual risk-adapted basis. LoE: high

The neuro-urological checks can be used as "gate-keepers" for paraplegiologic surveillance (bowel function, decubitus, spasticity, pain, etc.). LoE: high

Bladder cancer occurs more frequently and at a younger age in individuals with SCI and spina bifida compared to the able-bodied population. It tends to be more invasive and of higher grade, often involving squamous cell carcinomas (R. Böthig et al., 2021; Ralf Böthig, Domurath, Bremer, Vance, & Kaufmann, 2016). LoE: high

Recommendations:

- Regular evaluations of the bladder, renal tract, bladder function, and bladder management in individuals with SCI must be performed. GoR: A.
- Level of agreement: 100%
- Detailed recommendations can be found in: S2k Guideline of the German-Speaking Medical Society for Paraplegia (DMGP), AWMF Registry No.: 179/001: Neuro-urological diagnosis and therapy of lower urinary tract dysfunction in patients with spinal cord injury : S2k Guideline of the German-Speaking Medical Society of Paraplegia (DMGP), AWMF register no. 179/001*(R. Böthig et al., 2021).*

***8.2 Prevention of breast and cervical cancer (s620, s630, b640-b679)***

Compared to able-bodied women, women with SCI are less likely to participate in breast and cervical cancer screening programs (Lavela, Weaver, Smith, & Chen, 2006). This is concerning because non-participation has been associated with an increased prevalence of cervical cancer (R. Böthig et al., 2021). Especially in women with SCI above T6, who may have associated immune system dysfunctions, the risk of Human Papillomavirus (HPV)-associated dysplasia appears to be higher (Bais et al., 2007; Elenkov, Wilder, Chrousos, & Vizi, 2000; Nance & Sanders, 2007; Rove, Husmann, Wilcox, Vricella, & Higuchi, 2017). LoE: high

Recommendations:

- During annual SCI follow-up care, the importance of gynecological screening programs must be emphasized. GoR: A
- Women who have not been vaccinated for HPV should be informed about its relevance in preventing cervical cancer. GoR: B
- Women with SCI, especially those with limited hand function, a close follow-up by a gynecological specialist should be advised, as self-examination may not be feasible. GoR: B
- Level of agreement: 100%

***8.3 Sexual function (b640)***

Changes in sexual function significantly influence the Quality of Life (QOL) in individuals with SCI [188, 189]. It is reported that a significant percentage of men with sexual function problems are not adequately treated [2, 190]. Especially in individuals with spina bifida, sexual function, fertility, and pregnancy are often not adequately addressed (Dicianno et al., 2008; New, 2016; Simpson, Anwar, Wilson, & Bertapelle, 2006). LoE: high

Recommendations:

- During follow-up care, address fertility, sexual function, and sexuality, also in individuals with spina bifida. GoR: B
- As part of regular preventive examinations, inquiries about contraception and family planning should be made for all women with spinal cord injury in their fertile age. This is justified due to the need for optimal preparation for a planned pregnancy and the increased prevalence of risk factors for the development of venous thromboembolism under hormonal contraceptives. GoR: B
- Since unplanned pregnancies in women with spinal cord injury should be avoided due to the increased prevalence of pregnancy complications, efficient and safe contraception is necessary until the optimal time. Contraceptive counseling poses a challenge for women with spinal cord injury due to existing risk factors. It is essential to align the specific risk profile of the patient with the risk profile of contraceptive methods to make the optimal choice. GoR: B
- Level of agreement: 100%

Detailed recommendations can be found in: The S2k Guideline of the German-Speaking Medical Society for Paraplegia (DMGP), AWMF Registry No.: 179/001, Neuro-urological care of patients with spinal cord injury (R Böthig et al., 2017).

**Chapter 9: Contraception and peri-pregnancy management (b640, b660)**

***9.1 Contraceptives (b640 and b660)***

Women with SCI are at risk of developing Deep Vein Thrombosis (DVT) (Steven C Kirshblum, House, & O'Connor, 2002). The use of oral combined hormonal contraception is associated with an increased risk of DVT. The use of progestin monotherapy (except for Depot MedroxyProgesterone Acetate (DMPA)) can be considered for women at increased risk of DVT (Department of Reproductive Health, 2015; Kurze, Schmidt, Bertschy, Lange, & Kues, 2018; Petry et al., 1996; van Hylckama Vlieg, Helmerhorst, & Rosendaal, 2010; World Health Organization, 2015). Intrauterine systems (IUS) are also an alternative for women with SCI and can also be used during breastfeeding, in nulliparous women, and in women with a low risk of sexually transmitted diseases (STDs) (Centers for Disease Control and Prevention, 2016; Faculty of Sexual & Reproductive Healthcare, 2016). LoE: high

Because individuals with spina bifida have an increased risk of having a child with spina bifida, preventive folic acid intake is especially important in this group (Dicianno et al., 2008). Also in spina bifida, due to the risk of latex allergies, the use of latex-free contraceptives should be recommended (Spina Bifida Association, 2018). LoE: high

Recommendations:

- During annual follow-up, the need for gynecological referral, including discussions about contraception, family planning, and support before and during pregnancy should be assessed. GoR: B
  - The use of hormonal combination contraceptives in women with SCI must be avoided when possible due to the increased risk of venous thromboembolism (VTE). GoR: A
  - For women in stable relationships, progestin therapy or IUS should be considered as alternatives for contraception. GoR: B
  - In (young) women, the prevention of STDs should be discussed and appropriate methods such as condoms should be recommended. GoR: B
  - Latex-free contraceptives must be recommended for individuals with spina bifida. GoR: A
- Level of agreement: 100%

***9.2 Pregnancy, birth, and postpartum care (b640, b660)***

Due to the increased risk of complications, women with SCI and spina bifida should be regularly seen by interdisciplinary teams during pregnancy (Camune, 2013; Choi, Ji, & Han, 2017; Ghidini et al., 2008; Hughes, Short, Usherwood, & Tebbutt, 1991; Ines Kurze et al., 2018; Le Liepvre et al., 2017; Linstow et al., 2014; Morton et al., 2013; Pedaballe, Chhabra, Tandon, Chauhan, & Verma, 2018; Pereira, 2003; Sauer & Harvey, 1993; Sterling, Keunen, Wigdor, Sermer, & Maxwell, 2013). Regular follow-up appointments have been shown to reduce the number and severity of complications (Hughes et al., 1991). Before pregnancy, several aspects should be considered, including the use of folic acid, potential teratogenic medications, bladder management and its impact on pregnancy, weight management and sitting position, bowel management, risk of DVT, and prophylaxis LoE: moderate

Recommendations:

- Individuals with spina bifida should be informed about the risk of having children with spina bifida, recommend folic acid supplementation when pregnancy is planned, and refer for further counselling if needed. GoR: B
- Interdisciplinary and early referral to a gynecologist should be ensured for all women with planned pregnancies. GoR: B
- Medication use, DVT risk, aids, bladder and bowel management, and the risk of autonomic dysregulation should be regularly evaluated in all women with (planned) pregnancies. GoR: B
- Level of agreement: 100%

*See also:* S2k Guideline 179-002 "Pregnancy, childbirth, and the postpartum period in women with spinal cord injury" *(Ines Kurze et al., 2018)*

**Chapter 10: Musculoskeletal system**

The assessment of muscle strength and range of motion (ROM) are important evaluations along the continuum of care. Within the assessment of the musculoskeletal system, evaluation of spasticity (b735) should be an integral part. Spasticity / spastic syndrome is described in chapter 1.3

***10.1 Upper extremities (b7)***

Upper extremity problems are more common in persons with SCI and often appear at a younger age (Andretta et al., 2019; Erhan et al., 2013; Robertson, Dawood, & Ashworth, 2020). Individuals with tetraplegia, those dependent on wheelchairs, those with longer time since injury (TSI), the elderly, individuals with limited range of motion (ROM) in the upper extremities, and those with spasticity are at an increased risk of developing pain in the upper extremities, especially the shoulders (Ballinger, Rintala, & Hart, 2000; Curtis et al., 1995; Fullerton, Borckardt, & Alfano, 2003; Garofalo et al., 2011; van Drongelen et al., 2006), elbow (Erhan et al., 2013), as well as developing carpal tunnel syndrome (Alm, Saraste, & Norrbrink, 2008; van Drongelen et al., 2005). LoE: High

Recommendations:

- Annual evaluation and clinical examination of the upper extremities should be recommended for all persons with SCI, especially those with an increased risk profile. This evaluation should include: GoR: B.
  - Assessment of pain and its impact on activities and participation (VAS/NRS, WUSPI)
  - Evaluation of ROM and manual muscle strength, and muscle tone
  - Neurological assessement (ISNCSCI)
  - Assessment of aids including splints, walking sticks, rollators, wheelchairs, hand bike and E-support systems, as well as sitting position, transfer techniques, and wheelchair handling.
- In case of positive signs or symptoms, further evaluation should be considered (X-rays, ultrasound, artho-MRI, neurography). GoR: B
- Level of agreement: 100%

See also: AWMF guideline "Improvement of Upper Extremity Function in Cervical Spinal Cord Injury," Registration Number: 179-013, Development Stage: S2e. " (Akbar et al., 2013).

**10.2 Lower extremities (b7)**

Individuals with SCI and spina bifida are more likely to develop orthopedic problems, especially lower extremity contractures due to spasticity or lower extremity malformations (Diong, Harvey, et al., 2012; Sigrist-Nix et al., 2020; Yang et al., 2009). Contractures can negatively impact ambulatory performance, seating position in wheelchairs, and intimate care (Diong, Herbert, Kwah, Clarke, & Harvey, 2012; Swaroop & Dias, 2009). Early diagnosis of limitations in joint mobility is crucial for early intervention and the potential reversibility of contractures (McKinley, Santos, Meade, & Brooke, 2007). LoE: High

Recommendations:

- As part of lifelong follow-up care, regular or annual evaluation of potential upper extremity problems (specifically shoulder, elbow, wrist, and carpal tunnel syndrome) should be conducted for all individuals with SCI. GoR: B
- It is advisable to perform these evaluations for all patients, with a special emphasis on older individuals and those who have been in a wheelchair for an extended period. Upper extremity issues are more prevalent in all individuals with SCI, but those with tetraplegia, complete lesions, limited joint mobility, and spasticity are at a higher risk and should be particularly monitored. GoR: B

The evaluation should minimally include GoR: B:

- Pain history and its impact on daily activities (VAS/NRS, WUSPI, ISCoS Data Sets)
- Upper extremity joint status
- The International Standards for Neurological Classification of Spinal Cord Injury (ISNCSCI)
- Additional clinical examination as needed (muscle tone)
- Assessment of assistive devices and seating position
- Evaluation of transfers and wheelchair handling
- If there is a positive history and/or clinical examination, additional diagnostic tests should be considered. GoR: B
- Level of agreement: 100%

See also: " AWMF guideline "Rehabilitation of the Lower Extremities, Standing, and Walking Function in People with Spinal Cord Injury," Registration Number: 179-009, Development Stage: S2e (Dunteman, Vankoski, & Dias, 2000).

***10.3 Neurogenic scoliosis (s760)***

Approximately 10-21% of all adults with SCI develop scoliosis, especially those with tetraplegia, complete lesions, or spasticity (Harvey et al., 2017; Scheel-Sailer et al., 2018; Yagi et al., 2015). In children with SCI, it is reported that 100% develop scoliosis (Bergström, Short, Frankel, Henderson, & Jones, 1999), and in persons with spina bifida, percentages of 45% are reported depending on the neurological level (Kulshrestha et al., 2020; Lancourt, Dickson, & Carter, 1981). Scoliosis can lead to deterioration of sitting position, an increased risk of pressure injuries, pain, problems with bladder and bowel management, decreased mobility (especially in ambulatory individuals), and impaired cardiac and pulmonary function (Kulshrestha et al., 2020). LoE: high

Recommendations:

- A clinical evaluation of the spine (scoliosis and sagittal profile) should be conducted annually (more frequently if indicated), especially in persons with tetraplegia, complete lesions, adolescents (and those with spina bifida). GoR: B
- If the clinical evaluation suggests scoliosis/sagittal profile deterioration, supplementary evaluation is recommended (X-ray, function X-Ray, standing position for ambulatory individuals, sitting position for wheelchair-dependent individuals). GoR: B
- Wheelchairs, cushions, bandages, splints, and corsets should be evaluated annually (at least). GoR: B
- Level of agreement: 100%

**Chapter 11: Pressure injuries (s810, b810, b820)**

Pressure injuries develop due to pressure, friction, and shear forces (Meier et al., 2019; White et al., 2017) and are among the most severe SHCs in persons with SCI, associated with increased mortality (McDonnell & McCann, 2000). Pressure injuries in persons with SCI typically develop in areas such as the sacrum, os ileum, trochanter major, and heels, although other regions might also be affected (McDonnell & McCann, 2000). Risk factors for pressure injuries include complete injuries, vascular disease, malnutrition, dehydration, the use of certain medications (steroids), limited ROM, autonomic dysregulation, and cognitive limitations (McDonnell & McCann, 2000; White et al., 2017). Pressure injuries significantly impact quality of life (QOL) and result in substantial costs (Berven & Bradford, 2002; Garber & Rintala, 2003). Optimizing aids and sitting position are key factors in the prevention of pressure injuries (NICE & National Institute for Health and Care Excellence, 2014). LoE: high

Recommendations:

- Visual and palpatory evaluation of all areas at risk must be performed at every follow-up appointment. GoR: A
- Assessment of risk factors must be performed during annual follow-up appointments. GoR: A
- Evaluation of aids must be performed during annual follow-up appointments. GoR: A
- Regular (at least annual) evaluation of blood values (especially hematological, chemical including total protein, minerals, zinc, renal and liver function, Vitamin C, Selenium, Lipids, Vitamin B12, nutritional status) should be performed in all persons with SCI, especially those with recurrent pressure injuries and infections. GoR: B
- Level of agreement: 100%

See also AWMF Guideline "Treatment and prevention of pressure injury in spinal cord injury" Registration Number: 179-008, S1 (Biglari et al., 2017).

**Chapter 12: Psychological health (b130)**

The prevalence of depression in persons with SCI has been reported to range between 20% and 45% (Anderson, Vogel, Chlan, Betz, & McDonald, 2007; Hancock, Craig, Dickson, Chang, & Martin, 1993), and the prevalence of suicide is higher in persons with SCI compared to the able-bodied population (Gill, 1999; Hancock et al., 1993). Access to regular follow-up care has been associated with a lower risk of depression (Cuijpers, Dekker, Hollon, & Andersson, 2009). LoE: high

Recommendations:

- At every annual follow-up appointment, and especially in individuals with newly acquired SCI, those with tetraplegia, young persons, and those with other risk factors (such as pain or financial problems) for depression and psychological issues, screening for the presence of depressive symptoms must be performed. GoR: A
- Level of agreement: 100%

See also AWMF-Guideline "Depression in persons with spinal cord injury" Registration Number 179-003, S1 (Drzin-Schilling et al., 2016).

**Chapter 13: Medication and polypharmacy (e110)**

Polypharmacy is a common phenomenon in persons with SCI (Dunn, Love, & Ravesloot, 2000; Latimer, Ginis, Hicks, & McCartney, 2004). Medication is often incorrectly administered (Guilcher et al., 2018; Latimer et al., 2004), and side effects can lead to new health problems (e.g. constipation, changes in blood pressure, fatigue) (Cadel et al., 2019). It has been reported that when medication is regularly evaluated, the number of medications often can be reduced (Dunn et al., 2000). There is a lack of data on the use of non-prescribed medications; however, from clinical experience, this might not be irrelevant. LoE: moderate-high

Recommendations:

- As part of lifelong follow-up care, the medication list should be reviewed (and possibly adjusted) for indication, dosage (possibly adjusted for kidney function), side effects, and accuracy of intake. GoR: B
- Individuals should be informed about the indication, side effects, and intake of medications. GoR: B
- As part of lifelong follow-up care, consideration can be given to inquire about the intake of non-prescribed medications. GoR: 0
- Level of agreement: 100%

# Supplementary Figures and Tables

|  |  | **Control** | **Patient group** | **Description / potential assessment** | **AWMF Guidelines** |
| --- | --- | --- | --- | --- | --- |
| **Neurological** | **Anamnesis** | Motor skills, sensitivity | All | Changes in motor skills, sensitivity |  |
|  |  | Autonomic function | Alle, especially persons with lesion >T6 | Changes in autonomic function |  |
|  |  | spasticity | All | Changes in spasticity  PSFS | S2k 030-078 |
|  |  | (Neuropathic) Pain | All | Localisation / Quality  VAS oder NRS  ISCIP  SCIPI  Influence on activity, sleep and mood  QST | S2k 179-006  S2e-179-012 |
|  |  | Cognition | All, especially older persons, SB, SHT | MMSE  MoCA  Cognitive testing for spina bifida |  |
|  |  | Shunt problems | SB | Symptoms shunt dysfunction |  |
|  |  | sleep ex. sleep-related breathing disorder symptoms | All, especially Tetraplegia | ESS  Berlin-survey | S2k 030-045  S3 063-001 |
|  | **Clinical examination** | Neurological examination | All | ISNCSCI  Depth sensitivity | S2e 179-009  S2e-179-012 |
|  |  | Muscle status upper extremities | Especially Tetraplegia | ISNCSCI  MMT | S2e 179-013 |
|  |  | Muscle status lower extremities | All | ISNCSCI  MMT | S2e 179-009 |
|  |  | Walking and standing function | Ambulatory | 6 MWT  10 MWT  TUG  WISCI I / WISCI II  BBS  SCI-FAI | S2e 179-009 |
|  |  | Muscle reflexes and pathological reflexes | All | Reflex status |  |
|  |  | Spasticity | All | MAS  SCATS  SCI-SET  TS | S2k 030-078  S2e 179-009 |
|  |  | (Neuropathic) Pain | If pain | Nociceptive pain  ISNCSCI | S2k 179-006 |
|  |  | Changes in autonomic function | All |  |  |
|  | **Additional diagnostics** | Shunt control in case of clinical suspicion | SB | - |  |
|  |  | MRI-spinal cord, skull in case of clinical suspicion | All | - |  |
|  |  | Dysphagia | All, especially Tetraplegia | BODS  GUSS  FEES  DHI | S2e 179-012  S1 030-111 |

Table 1: Consensus-based paraplegiological control in the context of lifelong follow-up for people with spinal cord injury/disorder.

| F |  | **Control** | **Patient group** | **Description / potential assessment** | **AWMF Guidelines** |
| --- | --- | --- | --- | --- | --- |
| **General internal medicine** | **Anamnesis** | Medication | All | Current Medication |  |
|  |  | Diagnosis | All | New diagnosis |  |
|  |  | Participating in preventive care | All | Participating in preventive care |  |
|  |  | Quality of life | All | WHO-QOL BREF  WHO-QOL BREF 6 items  ISCoS QOL Data Set | S2e 179-012 |
|  |  | Mood | All | Self-assessment questionnaires should not ask about physical symptoms  HADS  DASS-21 | S1 179-003  S2e 179-012 |
|  |  | Sleep apnea symptoms | All, especially Tetraplegia | ESS  Berlin-Survey | S2k 179-011 |
|  |  | Cardiovascular anamnesis | All | Clinical symptoms |  |
|  |  | Pulmonary anamnesis | All, especially Tetraplegia | Clinical symptoms  Smoking status  Complications  Ventilation situation | S2k 179-011 |
|  |  | Vaccinations status | All, especially Tetraplegia | Vaccination status |  |
|  |  | Allergies | All, especially spina bifida | allergies |  |
|  |  | Fecal problems & bowel management | All | Method, time and frequency  Unsuccessful attempts  Obstipation  Incontinence  Pain  SCIM III  BSS  nDFS  ISAFSCI | S2k 179-004 |
|  |  | Hemorrhoids& Anal fissurs | All | Blood in stool | S2k 179-004 |
|  |  | Reflux | All | - |  |
|  |  | Pressure injuries | All, especially AIS A & B | NPIAP | S1 179-008  S2e 179-012 |
|  |  | Nutrition and lifestyle | All | Nutrition und drinking volume  Alcohol  Smoking  SNST | S2e 179-012 |
|  | **Clinical examination** | Blood pressure & heart frequency | All | Blood pressure  Heart frequency |  |
|  |  | Height, Weight | All | Weight  Height |  |
|  |  | Heart | All | Auskultation Herz |  |
|  |  | Vascular state | All | Vascular state |  |
|  |  | Lungs | All | Examination lungs |  |
|  |  | Abdomen | All | Examination abdomen |  |
|  |  | Skin | All | NPIAP  Localisation | S1 179-008 |
|  |  | Hemorrhoids & Anal fissurs | All | Hemorrhoids classification  Anal fissurs | S2k 179-004 |
|  | **Additional diagnostics** | Hemogram | All | Lipidprofile, Glucose, Hb1Ac |  |
|  |  | ECG | Tetraplegia (more than 10 years >TSI or 60 years) |  |  |
|  |  | Spirometry | Tetraplegia (maybe also > T12), older persons | FVC  FEV1  PEF  MIP  MEP  PIF  IVC | S2k 179-011  S2e 179-012 |
|  |  | Pulse oxymetrie | All, especially Tetraplegia |  |  |
|  |  | Polygraphy / Polysomnography | All, especially Tetraplegia |  |  |
|  |  | Ultrasound Abdomen | All | As part of the urological check-up, internal abdominal ultrasound |  |
|  |  | Abdominal overview scan | All | Maybe determination of colon transit time | S2k 179-004 |
|  |  | Video fluoroscopy | All, especially Tetraplegia |  |  |

|  |  | **Control** | **Patient group** | **Description / potential assessment** | **AWMF Guidelines** |
| --- | --- | --- | --- | --- | --- |
| **Neurological & urogenital** | **Anamnesis** | Bladder management | All | Methode, Häufigkeit  Inkontinenz  SCIM III  Qualiveen | S2K 179-001 |
|  |  | Infections | All | Prevalence  Duration, symptoms | S2K 179-001 |
|  |  | prevention | All | prevention | S2K 179-001 |
|  |  | Sexual function women & men | All | Sexual function  ISAFSCI | S2K 179-001 |
|  |  | Menstruation | Women | Menstruation | S2k 179-002 |
|  |  | Pregnancy and contraception | If indicated | - | S2k 179-002 |
|  | **Clinical examination** | Abdomen | All | Abdominal examination |  |
|  |  | Prostata (Urology) | Men | Rectal prostate examination (Urology) | S2K 179-001 |
|  |  | Prevention women (Gynecologist) | Women | Gynecological screening women (Gynecologist) | S2k 179-002 |
|  | **Additional diagnostics** | Kidney function | See guideline 179-001 | | S2K 179-001 |
|  |  | PSA | See guideline 179-001 | | S2K 179-001 |
|  |  | Urine analysis | See guideline 179-001 | | S2K 179-001 |
|  |  | Ultrasound kidney/bladder | See guideline 179-001 | | S2K 179-001 |
|  |  | Urodynamic examination | See guideline 179-001 | | S2K 179-001 |
|  |  | Cystoscopy | See guideline 179-001 | | S2K 179-001 |
| **Musculoskeletal** | **Anamnesis** | Pain musculoskeletal system | All, wheelchair users | VAS/NRS  WUSPI | S2k 179-006 |
|  |  | Joint mobility / stability | Alle, wheelchair users | Joint mobility / stability | S2e 179-009 |
|  |  | Fractures | All | - | S1 179-007 |
|  |  | Change seating position | All, wheelchair users | Seating position |  |
|  |  | Orthosis | If indicated | - |  |
|  |  | Mobility | All | SCIM |  |
|  | **Clinical examination** | Upper extremity | All, especially tetraplegia, wheelchair users | Joint status upper extremities |  |
|  |  | Arm- handfunction | Tetraplegia | GRASSP (Teil)  ICSHT classifikation  Jamar  GRT  VLT-SV  CUE-Q  Duruöz Hand Index  Jebsen Hand Function Test Box and Blocks Test  Hand Held Myometer | S2e 179-013  S2e 179-012 |
|  |  | Shoulder | Wheelchair users and walking aids | Shoulder examination |  |
|  |  | Lower extremities | All, especially wheelchair users | Joint status lower extremities |  |
|  |  | Scoliosis / Seating position | All, especially wheelchair users | Scoliosis / seating position |  |
|  |  | Mobility / standing / Walking | Whellchair users, persons able to walk | Wheelchair course  Walking  Functional testing (Berg Balance Test, Time-up & go Test, etc) | S2e 179-009 |
|  | **Additional examination** | X-spine (in seating or standing) | All, especially tetraplegia, wheelchair users | X-spine |  |
|  |  | Osteoporosis | All, especially whellchair users | DX Osteodensitometry or peripheral quantitative computed tomography Determination of serum calcium and 25-hydroxyvitamin D3 | S1 179-007 |
|  |  | Gait analysis | Persons able to walk | Gait analysis |  |

|  |  | **Control** | **Patient group** | **Description / potential assessment** | **AWMF Guidelines** |
| --- | --- | --- | --- | --- | --- |
| **Rehabilitation status** | **Anamnesis** | Mobility (in house, out of house) | All | SCIM III  WISCI II | S2e 179-009  S2e 179-012 |
|  |  | Independence & assistance / Support / Nursing care | All | SCIM III |  |
|  |  | Stress / overload on relatives | All |  |  |
|  |  | Wheelchair / assistive devices /Orthosis | All | AMR | S2e 179-012 |
|  |  | Therapies (Frequency / Content / goals) | All | - |  |
|  |  | Sociale situation | All | - |  |
|  |  | Living situation | All | - |  |
|  |  | School / Education & professional situation | All | PASIPD  WORQ-Self | S2e 179-012 |
|  |  | Hobbies | All | PASIPD |  |
|  |  | Insurance state | All | - |  |

**
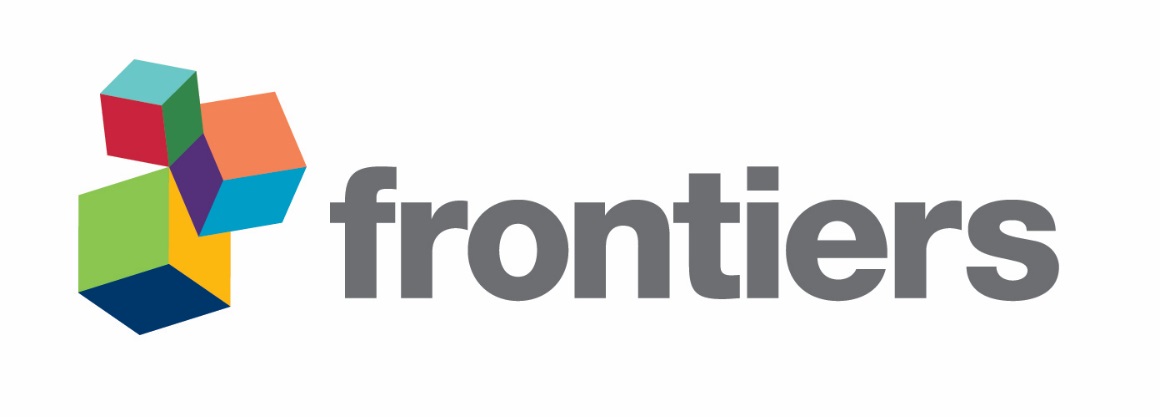
**

**References**

Akbar, M., Penzkofer, S., Weber, M. A., Bruckner, T., Winterstein, M., & Jung, M. (2013). Prevalence of carpal tunnel syndrome and wrist osteoarthritis in long-term paraplegic patients compared with controls. *Journal of Hand Surgery (European Volume), 39*(2), 132-138. doi:10.1177/1753193413478550

Ali, H. B., Preci Hamilton, S. Z., & Yakoub, K. M. (2018). Spinal arachnoid web—a review article. *Journal of Spine Surgery, 4*(2), 446. Retrieved from <http://jss.amegroups.com/article/view/4168/4738>

Alm, M., Saraste, H., & Norrbrink, C. (2008). Shoulder Pain in Persons with Thoracic Spinal Cord Injury: Prevalence and Characteristics. *Journal of Rehabilitation Medicine, 40*(4), 277-283. doi:10.2340/16501977-0173

Anderson, C. J., Vogel, L. C., Chlan, K. M., Betz, R., & McDonald, C. M. (2007). Depression in Adults Who Sustained Spinal Cord Injuries as Children or Adolescents. *The Journal of Spinal Cord Medicine, 30*(sup1), S76-S82.

Andretta, E., Landi, L. M., Cianfrocca, M., Manassero, A., Risi, O., & Artuso, G. (2019). Bladder management during pregnancy in women with spinal-cord injury: an observational, multicenter study. *International Urogynecology Journal, 30*(2), 293-300. doi:10.1007/s00192-018-3620-8

Ausili, E., Tabacco, F., Focarelli, B., Nucera, E., Patriarca, G., & Rendeli, C. (2007). Prevalence of latex allergy in spina bifida: genetic and environmental risk factors. *Eur Rev Med Pharmacol Sci, 11*(3), 149-153.

Azarbal, A., Rowell, S., Lewis, J., Urankar, R., Moseley, S., Landry, G., & Moneta, G. (2011). Duplex ultrasound screening detects high rates of deep vein thromboses in critically ill trauma patients. *J Vasc Surg, 54*(3), 743-747; discussion 747-748. doi:10.1016/j.jvs.2011.02.058

Bais, A. G., Van Kemenade, F. J., Berkhof, J., Verheijen, R. H., Snijders, P. J., Voorhorst, F., . . . Meijer, C. J. (2007). Human papillomavirus testing on self‐sampled cervicovaginal brushes: an effective alternative to protect nonresponders in cervical screening programs. *International journal of cancer, 120*(7), 1505-1510.

Ballinger, D. A., Rintala, D. H., & Hart, K. A. (2000). The relation of shoulder pain and range-of-motion problems to functional limitations, disability, and perceived health of men with spinal cord injury: a multifaceted longitudinal study. *Arch Phys Med Rehabil, 81*(12), 1575-1581. doi:10.1053/apmr.2000.18216

Banerjea, R., Sambamoorthi, U., Weaver, F., Maney, M., Pogach, L. M., & Findley, T. (2008). Risk of stroke, heart attack, and diabetes complications among veterans with spinal cord injury. *Archives of Physical Medicine and Rehabilitation, 89*(8), 1448-1453.

Bauman, W. A., & Cardozo, C. P. (2015). Osteoporosis in individuals with spinal cord injury. *PM R, 7*(2), 188-201; quiz 201. doi:10.1016/j.pmrj.2014.08.948

Bauman, W. A., Cirnigliaro, C. M., La Fountaine, M. F., Martinez, L., Kirshblum, S. C., & Spungen, A. M. (2015). Zoledronic acid administration failed to prevent bone loss at the knee in persons with acute spinal cord injury: an observational cohort study. *J Bone Miner Metab, 33*(4), 410-421. doi:10.1007/s00774-014-0602-x

BeDell, K. K., Scremin, A. M., Perell, K. L., & Kunkel, C. F. (1996). Effects of functional electrical stimulation-induced lower extremity cycling on bone density of spinal cord-injured patients. *Am J Phys Med Rehabil, 75*(1), 29-34. doi:10.1097/00002060-199601000-00008

Beerepoot, M. A., Geerlings, S. E., van Haarst, E. P., van Charante, N. M., & ter Riet, G. (2013). Nonantibiotic prophylaxis for recurrent urinary tract infections: a systematic review and meta-analysis of randomized controlled trials. *J Urol, 190*(6), 1981-1989. doi:10.1016/j.juro.2013.04.142

Bergström, E. M., Short, D. J., Frankel, H. L., Henderson, N. J., & Jones, P. R. (1999). The effect of childhood spinal cord injury on skeletal development: a retrospective study. *Spinal Cord, 37*(12), 838-846. doi:10.1038/sj.sc.3100928

Berlowitz, D. J., Brown, D. J., Campbell, D. A., & Pierce, R. J. (2005). A longitudinal evaluation of sleep and breathing in the first year after cervical spinal cord injury. *Archives of Physical Medicine and Rehabilitation, 86*(6), 1193-1199.

Berven, S., & Bradford, D. S. (2002). Neuromuscular scoliosis: causes of deformity and principles for evaluation and management. *Semin Neurol, 22*(2), 167-178. doi:10.1055/s-2002-36540

Biglari, B., Dissemond, J., Donhauser, M., Föcks, I., Fürstenberg, C. H., Gstaltner, K., . . . Ziegler, R. (2017). *Querschnittspezifische Dekubitusbehandlung und -prävention*. Retrieved from

Billington, Z. J., Henke, A. M., & Gater, D. R., Jr. (2022). Spasticity Management after Spinal Cord Injury: The Here and Now. *J Pers Med, 12*(5). doi:10.3390/jpm12050808

Bjelakovic, B., Dimitrijevic, L., Lukic, S., & Golubovic, E. (2014). Hypertensive encephalopathy as a late complication of autonomic dysreflexia in a 12-year-old boy with a previous spinal cord injury. *European journal of pediatrics, 173*(12), 1683-1684. Retrieved from <https://link.springer.com/article/10.1007/s00431-014-2281-y>

Blok, B., Castro-Diaz, D., Del Popolo, G., Groen , J., Hamid, R., Karsenty, G., . . . Pannek, J. (2019). *EAU Guidelines on Neuro-Urology* Retrieved from <https://uroweb.org/guidelines/neuro-urology>

Bonfield, C. M., Levi, A. D., Arnold, P. M., & Okonkwo, D. O. (2010). Surgical management of post-traumatic syringomyelia. *Spine (Phila Pa 1976), 35*(21 Suppl), S245-258. doi:10.1097/BRS.0b013e3181f32e9c

Böthig, R., Burkhard, D., Kaufmann, A., Bremer, J., Will, V., & Kirschner-Hermanns, R. (2021). *S2k-Leitlinie der Deutschsprachigen Medizinischen Gesellschaft für Paraplegie (DMGP). Neuro-urologische Versorgung querschnittgelähmter Patienten.* . Retrieved from

Böthig, R., Domurath, B., Bremer, J., Vance, W., & Kaufmann, A. (2016). Neuro-urologische Versorgung querschnittgelähmter Patienten. In.

Böthig, R., Domurath, B., Kaufmann, A., Bremer, J., Vance, W., & Kurze, I. (2017). Neuro-urological diagnosis and therapy of lower urinary tract dysfunction in patients with spinal cord injury: S2k Guideline of the German-Speaking Medical Society of Paraplegia (DMGP), AWMF register no. 179/001. *Der Urologe. Ausg. A, 56*(6), 785-792. Retrieved from <https://link.springer.com/article/10.1007%2Fs00120-017-0354-z>

Bowman, R. M., McLone, D. G., Grant, J. A., Tomita, T., & Ito, J. A. (2001). Spina bifida outcome: a 25-year prospective. *Pediatr Neurosurg, 34*(3), 114-120. doi:10.1159/000056005

Brinkhof, M. W., Al-Khodairy, A., Eriks-Hoogland, I., Fekete, C., Hinrichs, T., Hund-Georgiadis, M., . . . Swi, S. C. I. S. G. (2016). Health conditions in people with spinal cord injury: Contemporary evidence from a population-based community survey in Switzerland. *J Rehabil Med, 48*(2), 197-209. doi:10.2340/16501977-2039

Brommer, B., Engel, O., Kopp, M. A., Watzlawick, R., Muller, S., Pruss, H., . . . Schwab, J. M. (2016). Spinal cord injury-induced immune deficiency syndrome enhances infection susceptibility dependent on lesion level. *Brain, 139*(Pt 3), 692-707. doi:10.1093/brain/awv375

Brown, R., DiMarco, A. F., Hoit, J. D., & Garshick, E. (2006). Respiratory dysfunction and management in spinal cord injury. *Respir Care, 51*(8), 853-868;discussion 869-870.

Bubbear, J. S., Gall, A., Middleton, F. R., Ferguson-Pell, M., Swaminathan, R., & Keen, R. W. (2011). Early treatment with zoledronic acid prevents bone loss at the hip following acute spinal cord injury. *Osteoporos Int, 22*(1), 271-279. doi:10.1007/s00198-010-1221-6

Budd, M. A., Dixon, T. M., Barnett, S. D., Njoh, E., Goetz, L. L., & Ottomanelli, L. (2017). Examination of traumatic brain injury exposure among veterans with spinal cord injury. *Rehabil Psychol, 62*(3), 345-352. doi:10.1037/rep0000129

Burns, A. S., St-Germain, D., Connolly, M., Delparte, J. J., Guindon, A., Hitzig, S. L., & Craven, B. C. (2015). Phenomenological study of neurogenic bowel from the perspective of individuals living with spinal cord injury. *Archives of Physical Medicine and Rehabilitation, 96*(1), 49-55. e41.

Buzzell, A., Chamberlain, J. D., Eriks-Hoogland, I., Hug, K., Jordan, X., Schubert, M., . . . the Swiss National, C. (2020). All-cause and cause-specific mortality following non-traumatic spinal cord injury: evidence from a population-based cohort study in Switzerland. *Spinal Cord, 58*(2), 157-164. doi:10.1038/s41393-019-0361-6

Buzzell, A., Chamberlain, J. D., Schubert, M., Mueller, G., Berlowitz, D. J., & Brinkhof, M. W. G. (2020). Perceived sleep problems after spinal cord injury: Results from a community-based survey in Switzerland. *J Spinal Cord Med*, 1-10. doi:10.1080/10790268.2019.1710938

Cadel, L., A, C. E., Hitzig, S. L., Packer, T. L., Patel, T., Lofters, A., & Guilcher, S. J. T. (2019). Spinal cord injury and polypharmacy: a scoping review. *Disabil Rehabil*, 1-13. doi:10.1080/09638288.2019.1610085

Cameron, A. P., Rodriguez, G. M., & Schomer, K. G. (2012). Systematic review of urological followup after spinal cord injury. *The Journal of urology, 187*(2), 391-397. Retrieved from <https://www.sciencedirect.com/science/article/abs/pii/S0022534711052645?via%3Dihub>

Camune, B. D. (2013). Challenges in the management of the pregnant woman with spinal cord injury. *J Perinat Neonatal Nurs, 27*(3), 225-231. doi:10.1097/JPN.0b013e31829ca83f

Carroll, A. M., & Brackenridge, P. (2005). Post-traumatic syringomyelia: a review of the cases presenting in a regional spinal injuries unit in the north east of England over a 5-year period. *Spine (Phila Pa 1976), 30*(10), 1206-1210. doi:10.1097/01.brs.0000162277.76012.0b

Castro, M. M., & Daltro, C. (2009). Sleep patterns and symptoms of anxiety and depression in patients with chronic pain. *Arq Neuropsiquiatr, 67*(1), 25-28. doi:10.1590/s0004-282x2009000100007

Centers for Disease Control and Prevention. (2016). *U.S: Medical Eligibility Creiteria for contraceptive Use*. Retrieved from

Choi, E. K., Ji, Y., & Han, S. W. (2017). Sexual function and quality of life in young men with spina bifida: could it be neglected aspects in clinical practice? *Urology, 108*, 225-232. Retrieved from <https://www.sciencedirect.com/science/article/pii/S0090429517301644?via%3Dihub>

Clark, J. M., Jelbart, M., Rischbieth, H., Strayer, J., Chatterton, B., Schultz, C., & Marshall, R. (2007). Physiological effects of lower extremity functional electrical stimulation in early spinal cord injury: lack of efficacy to prevent bone loss. *Spinal Cord, 45*(1), 78-85. doi:10.1038/sj.sc.3101929

Consortium for Spinal Cord Medicine, & Paralyzed Veterans of America. (2014). *Pressure Ulcer Prevention and Treatment Following Spinal Cord Injury: A Clinial Pracitce Guideline for Health-Care Professionals*. Retrieved from Washington, DC:

Cragg, J. J., Ravensbergen, H. J., Borisoff, J. F., & Claydon, V. E. (2015). Optimal scaling of weight and waist circumference to height for adiposity and cardiovascular disease risk in individuals with spinal cord injury. *Spinal Cord, 53*(1), 64-68. doi:10.1038/sc.2014.165

Cuijpers, P., Dekker, J., Hollon, S. D., & Andersson, G. (2009). Adding psychotherapy to pharmacotherapy in the treatment of depressive disorders in adults: a meta-analysis. *J Clin Psychiatry, 70*(9), 1219-1229. doi:10.4088/JCP.09r05021

Curtis, K. A., Roach, K. E., Applegate, E. B., Amar, T., Benbow, C. S., Genecco, T. D., & Gualano, J. (1995). Reliability and validity of the Wheelchair User's Shoulder Pain Index (WUSPI). *Paraplegia, 33*(10), 595-601. doi:10.1038/sc.1995.126

Delcò, F., & Sonnenberg, A. (1998). Associations between hemorrhoids and other diagnoses. *Diseases of the colon and rectum, 41*(12), 1534-1541; discussion 1541-1532. doi:10.1007/bf02237302

Department of Reproductive Health, W. H. O. (2015). *Medical eligibility criteria for contraceptive use. Fifth edition*. Retrieved from

Dermanovic Dobrota, V., Hrabac, P., Skegro, D., Smiljanic, R., Dobrota, S., Prkacin, I., . . . Basic Kes, V. (2014). The impact of neuropathic pain and other comorbidities on the quality of life in patients with diabetes. *Health Qual Life Outcomes, 12*, 171. doi:10.1186/s12955-014-0171-7

Dicianno, B. E., Kurowski, B. G., Yang, J. M., Chancellor, M. B., Bejjani, G. K., Fairman, A. D., . . . Sotirake, J. (2008). Rehabilitation and medical management of the adult with spina bifida. *Am J Phys Med Rehabil, 87*(12), 1027-1050. doi:10.1097/PHM.0b013e31818de070

Dijkers, M., Bryce, T., & Zanca, J. (2009). Prevalence of chronic pain after traumatic spinal cord injury: a systematic review. *J Rehabil Res Dev, 46*(1), 13-29.

Diong, J., Harvey, L. A., Kwah, L. K., Eyles, J., Ling, M. J., Ben, M., & Herbert, R. D. (2012). Incidence and predictors of contracture after spinal cord injury--a prospective cohort study. *Spinal Cord, 50*(8), 579-584. doi:10.1038/sc.2012.25

Diong, J., Herbert, R. D., Kwah, L. K., Clarke, J. L., & Harvey, L. A. (2012). Mechanisms of increased passive compliance of hamstring muscle-tendon units after spinal cord injury. *Clin Biomech (Bristol, Avon), 27*(9), 893-898. doi:10.1016/j.clinbiomech.2012.07.003

Dionyssiotis, Y. (2012). Malnutrition in spinal cord injury: more than nutritional deficiency. *Journal of clinical medicine research, 4*(4), 227. Retrieved from <https://www.ncbi.nlm.nih.gov/pmc/articles/PMC3409617/pdf/jocmr-04-227.pdf>

Doubeni, C. A., Corley, D. A., Quinn, V. P., Jensen, C. D., Zauber, A. G., Goodman, M., . . . Zhao, W. K. (2018). Effectiveness of screening colonoscopy in reducing the risk of death from right and left colon cancer: a large community-based study. *Gut, 67*(2), 291-298. Retrieved from <https://gut.bmj.com/content/gutjnl/67/2/291.full.pdf>

Drzin-Schilling, B., Eisenhuth, J., Janker, P., Neikes, M., Prang, P., & Stolle, A. (2016). *S1-Leitlinie der Deutschsprachigen Medizinischen Gesellschaft für Paraplegie (DMPGP). Depression bei Menschen mit Querschnittlähmung: Besonderheiten in der Diagnostik und Behandlung*. Retrieved from

Duchac, S. (2016). Veränderung der Schluckphysiologie nach ventraler und dorsaler Operation an der Halswirbelsäule: eine retrospektive Vergleichsstudie.

Dudley-Javoroski, S., Petrie, M. A., McHenry, C. L., Amelon, R. E., Saha, P. K., & Shields, R. K. (2016). Bone architecture adaptations after spinal cord injury: impact of long-term vibration of a constrained lower limb. *Osteoporos Int, 27*(3), 1149-1160. doi:10.1007/s00198-015-3326-4

Dunn, M., Love, L., & Ravesloot, C. (2000). Subjective health in spinal cord injury after outpatient healthcare follow-up. *Spinal Cord, 38*(2), 84-91. doi:10.1038/sj.sc.3100957

Dunteman, R. C., Vankoski, S. J., & Dias, L. S. (2000). Internal derotation osteotomy of the tibia: pre- and postoperative gait analysis in persons with high sacral myelomeningocele. *J Pediatr Orthop, 20*(5), 623-628. doi:10.1097/00004694-200009000-00014

Dziewas, R. P., C. (2020). *Neurogene Dysphagie, S1-Leitlinie.* Retrieved from

el Masry, W. S., & Biyani, A. (1996). Incidence, management, and outcome of post-traumatic syringomyelia. In memory of Mr Bernard Williams. *J Neurol Neurosurg Psychiatry, 60*(2), 141-146. doi:10.1136/jnnp.60.2.141

Elenkov, I. J., Wilder, R. L., Chrousos, G. P., & Vizi, E. S. (2000). The sympathetic nerve—an integrative interface between two supersystems: the brain and the immune system. *Pharmacological reviews, 52*(4), 595-638.

Emmanuel, A. (2010). Rehabilitation in practice: Managing neurogenic bowel dysfunction. *Clinical rehabilitation, 24*(6), 483-488. Retrieved from <https://journals.sagepub.com/doi/pdf/10.1177/0269215509353253>

Erhan, B., Gündüz, B., Bardak, A. N., Özcan, S., Çarlı, A., Er, H., . . . Özçakar, L. (2013). Elbow problems in paraplegic spinal cord injured patients: frequency and related risk factors—a preliminary controlled study. *Spinal Cord, 51*(5), 406-408. doi:10.1038/sc.2013.13

Eriks-Hoogland, I., Hilfiker, R., Baumberger, M., Balk, S., Stucki, G., & Perret, C. (2011). Clinical assessment of obesity in persons with spinal cord injury: validity of waist circumference, body mass index, and anthropometric index. *The Journal of Spinal Cord Medicine, 34*(4), 416-422. doi:10.1179/2045772311Y.0000000014

Faaborg, P., Christensen, P., Krassioukov, A., Laurberg, S., Frandsen, E., & Krogh, K. (2014). Autonomic dysreflexia during bowel evacuation procedures and bladder filling in subjects with spinal cord injury. *Spinal Cord, 52*(6), 494-498. Retrieved from <https://www.nature.com/articles/sc201445.pdf>

Faculty of Sexual & Reproductive Healthcare. (2016). *UK Medical Eligibility Criteria for Contraceptive Use*. Retrieved from

Flavin, K., Ando, Y., & Teraoka, J. (2009). Autonomic dysreflexia caused by anal fissure. *PM R, 1*(10), 975-976. doi:10.1016/j.pmrj.2009.09.007

Flueck, J. L., & Perret, C. (2017). Vitamin D deficiency in individuals with a spinal cord injury: a literature review. *Spinal Cord, 55*(5), 428-434. doi:10.1038/sc.2016.155

Fullerton, H. D., Borckardt, J. J., & Alfano, A. P. (2003). Shoulder Pain: A Comparison of Wheelchair Athletes and Nonathletic Wheelchair Users. *Medicine & Science in Sports & Exercise, 35*(12). Retrieved from <https://journals.lww.com/acsm-msse/Fulltext/2003/12000/Shoulder_Pain__A_Comparison_of_Wheelchair_Athletes.2.aspx>

Garber, S. L., & Rintala, D. H. (2003). Pressure ulcers in veterans with spinal cord injury: a retrospective study. *J Rehabil Res Dev, 40*(5), 433-441. doi:10.1682/jrrd.2003.09.0433

Garofalo, R., Conti, M., Massazza, G., Cesari, E., Vinci, E., & Castagna, A. (2011). Subcoracoid impingement syndrome: a painful shoulder condition related to different pathologic factors. *Musculoskelet Surg, 95 Suppl 1*, S25-29. doi:10.1007/s12306-011-0142-7

Gastroenterologie, S. G. f. (2013). *Wegleitung Koloskopie SGG/SSG*. Retrieved from

Gater Jr, D. R. (2007). Obesity after spinal cord injury. *Physical medicine and rehabilitation clinics of North America, 18*(2), 333-351.

Geng, V., Böthing, R., Kurze, I., Hildesheim, A., & Leder Eckhart, D. (2019). *S2k-Leitlinie Neurogene Darmfunktionsstörung bei Querschnittlähmung.* . Retrieved from

Ghidini, A., Healey, A., Andreani, M., & Simonson, M. R. (2008). Pregnancy and women with spinal cord injuries. *Acta Obstet Gynecol Scand, 87*(10), 1006-1010. doi:10.1080/00016340802356909

Giangregorio, L. M., Hicks, A. L., Webber, C. E., Phillips, S. M., Craven, B. C., Bugaresti, J. M., & McCartney, N. (2005). Body weight supported treadmill training in acute spinal cord injury: impact on muscle and bone. *Spinal Cord, 43*(11), 649-657. doi:10.1038/sj.sc.3101774

Gill, M. (1999). Psychosocial Implications of Spinal Cord Injury. *Critical Care Nursing Quarterly, 22*(2), 1-7. Retrieved from <https://journals.lww.com/ccnq/Fulltext/1999/08000/Psychosocial_Implications_of_Spinal_Cord_Injury.2.aspx>

Goemaere, S., Van Laere, M., De Neve, P., & Kaufman, J. M. (1994). Bone mineral status in paraplegic patients who do or do not perform standing. *Osteoporos Int, 4*(3), 138-143. doi:10.1007/BF01623058

Goenka, S., Sethi, S., Pandey, N., Joshi, M., & Jindal, R. (2018). Effect of early treatment with zoledronic acid on prevention of bone loss in patients with acute spinal cord injury: a randomized controlled trial. *Spinal Cord, 56*(12), 1207-1211. doi:10.1038/s41393-018-0195-7

Goktepe, A. S., Tugcu, I., Yilmaz, B., Alaca, R., & Gunduz, S. (2008). Does standing protect bone density in patients with chronic spinal cord injury? *J Spinal Cord Med, 31*(2), 197-201. doi:10.1080/10790268.2008.11760712

Gore, M., Brandenburg, N. A., Dukes, E., Hoffman, D. L., Tai, K. S., & Stacey, B. (2005). Pain severity in diabetic peripheral neuropathy is associated with patient functioning, symptom levels of anxiety and depression, and sleep. *J Pain Symptom Manage, 30*(4), 374-385. doi:10.1016/j.jpainsymman.2005.04.009

Gorgey, A. S., Wells, K. M., & Austin, T. L. (2015). Adiposity and spinal cord injury. *World J Orthop, 6*(8), 567-576. doi:10.5312/wjo.v6.i8.567

Gormsen, L., Rosenberg, R., Bach, F. W., & Jensen, T. S. (2010). Depression, anxiety, health-related quality of life and pain in patients with chronic fibromyalgia and neuropathic pain. *Eur J Pain, 14*(2), 127.e121-128. doi:10.1016/j.ejpain.2009.03.010

Guilcher, S. J. T., Hogan, M. E., Calzavara, A., Hitzig, S. L., Patel, T., Packer, T., & Lofters, A. K. (2018). Prescription drug claims following a traumatic spinal cord injury for older adults: a retrospective population-based study in Ontario, Canada. *Spinal Cord, 56*(11), 1059-1068. doi:10.1038/s41393-018-0174-z

Gustorff, B., Dorner, T., Likar, R., Grisold, W., Lawrence, K., Schwarz, F., & Rieder, A. (2008). Prevalence of self‐reported neuropathic pain and impact on quality of life: A prospective representative survey. *Acta Anaesthesiologica Scandinavica, 52*(1), 132-136. Retrieved from <https://onlinelibrary.wiley.com/doi/abs/10.1111/j.1399-6576.2007.01486.x>

Han, S. J., Kim, C. M., Lee, J. E., & Lee, T. H. (2009). Colonoscopic lesions in patients with spinal cord injury. *The Journal of Spinal Cord Medicine, 32*(4), 404-407. doi:10.1080/10790268.2009.11753183

Hancock, K., Craig, A., Dickson, H., Chang, E., & Martin, J. (1993). Anxiety and depression over the first year of spinal cord injury: a longitudinal study. *Spinal Cord, 31*(6), 349-357.

Harari, D., & Minaker, K. (2000). Megacolon in patients with chronic spinal cord injury. *Spinal Cord, 38*(6), 331-339. Retrieved from <https://www.nature.com/articles/3101010.pdf>

Harvey, L. A., Katalinic, O. M., Herbert, R. D., Moseley, A. M., Lannin, N. A., & Schurr, K. (2017). Stretch for the treatment and prevention of contractures. *Cochrane Database Syst Rev, 1*, CD007455. doi:10.1002/14651858.CD007455.pub3

Hayashi, T., Fujiwara, Y., Sakai, H., Kubota, K., Kawano, O., Mori, E., . . . Maeda, T. (2020). The time course of dysphagia following traumatic cervical spinal cord injury: a prospective cohort study. *Spinal Cord, 58*(1), 53-57. doi:10.1038/s41393-019-0347-4

Hayman, A. V., Guihan, M., Fisher, M. J., Murphy, D., Anaya, B. C., Parachuri, R., . . . Bentrem, D. J. (2013). Colonoscopy is high yield in spinal cord injury. *J Spinal Cord Med, 36*(5), 436-442. doi:10.1179/2045772313y.0000000091

Hughes, S. J., Short, D. J., Usherwood, M. M., & Tebbutt, H. (1991). Management of the pregnant woman with spinal cord injuries. *Br J Obstet Gynaecol, 98*(6), 513-518. doi:10.1111/j.1471-0528.1991.tb10361.x

Jiang, S. D., Dai, L. Y., & Jiang, L. S. (2006). Osteoporosis after spinal cord injury. *Osteoporos Int, 17*(2), 180-192. doi:10.1007/s00198-005-2028-8

Kalke, Y.-B., Frotzer, A., Moosburger, J., & Wittgruber, G. (2018). *Querschnittlähmungsassoziierte Osteoporose*. Retrieved from

Karlsson, A. (1999). Autonomic dysreflexia. *Spinal Cord, 37*(6), 383-391.

Kheder, A., & Nair, K. P. S. (2012). Spasticity: pathophysiology, evaluation and management. *Practical Neurology, 12*(5), 289-298. doi:10.1136/practneurol-2011-000155

Kirshblum, S. C., Burns, S. P., Biering-Sorensen, F., Donovan, W., Graves, D. E., Jha, A., . . . Waring, W. (2011). International standards for neurological classification of spinal cord injury (revised 2011). *J Spinal Cord Med, 34*(6), 535-546. doi:10.1179/204577211x13207446293695

Kirshblum, S. C., House, J. G., & O'Connor, K. C. (2002). Silent autonomic dysreflexia during a routine bowel program in persons with traumatic spinal cord injury: a preliminary study. *Archives of Physical Medicine and Rehabilitation, 83*(12), 1774-1776. Retrieved from <https://www.sciencedirect.com/science/article/abs/pii/S0003999302006019?via%3Dihub>

Klekamp, J. (2012). Treatment of posttraumatic syringomyelia. *J Neurosurg Spine, 17*(3), 199-211. doi:10.3171/2012.5.Spine11904

Krassioukov, A. V., Furlan, J. C., & Fehlings, M. G. (2003). Autonomic dysreflexia in acute spinal cord injury: an under-recognized clinical entity. *Journal of neurotrauma, 20*(8), 707-716. Retrieved from <https://www.liebertpub.com/doi/pdfplus/10.1089/089771503767869944>

Krassioukov, A. V., Karlsson, A. K., Wecht, J. M., Wuermser, L. A., Mathias, C. J., Marino, R. J., . . . International Spinal Cord, S. (2007). Assessment of autonomic dysfunction following spinal cord injury: rationale for additions to International Standards for Neurological Assessment. *J Rehabil Res Dev, 44*(1), 103-112. doi:10.1682/jrrd.2005.10.0159

Krebs, J., Koch, H. G., Hartmann, K., & Frotzler, A. (2016). The characteristics of posttraumatic syringomyelia. *Spinal Cord, 54*(6), 463-466. doi:10.1038/sc.2015.218

Krogh, K., Christensen, P., Sabroe, S., & Laurberg, S. (2006). Neurogenic bowel dysfunction score. *Spinal Cord, 44*(10), 625-631. Retrieved from <https://www.nature.com/articles/3101887.pdf>

Kulshrestha, R., Kuiper, J. H., Masri, W. E., Chowdhury, J. R., Kaur, S., Kumar, N., . . . Osman, A. E. (2020). Scoliosis in paediatric onset spinal cord injuries. *Spinal Cord*. doi:10.1038/s41393-020-0418-6

Kumagai, G., Wada, K., Kudo, H., Asari, T., Ichikawa, N., & Ishibashi, Y. (2020). D-dimer monitoring combined with ultrasonography improves screening for asymptomatic venous thromboembolism in acute spinal cord injury. *J Spinal Cord Med, 43*(3), 353-357. doi:10.1080/10790268.2018.1518765

Kurze, I., Schmidt, M., Bertschy, S., Lange, U., & Kues, S. (2018). *S2k-Leitlinie 179-002. Schwangerschaft, Geburt und Wochenbett bei Frauen mit Querschnittlähmung*. Retrieved from

Kurze, I., Schmidt, M., Bertschy, S., Lange, U., Kues, S., & Fiebag, K. (2018). *Schwangerschaft, Geburt und Wochenbett bei Frauen mit Querschnittlähmung (Langfassung). S2k-Leitlinie.* Retrieved from

Lancourt, J., Dickson, J., & Carter, R. (1981). Paralytic spinal deformity following traumatic spinal-cord injury in children and adolescents. *The Journal of bone and joint surgery. American volume, 63*(1), 47-53.

Latimer, A., Ginis, K., Hicks, A., & McCartney, N. (2004). An examination of the mechanisms of exercise-induced change in psychological well-being among people with spinal cord injury. *Journal of rehabilitation research and development, 41*, 643-652. doi:10.1682/JRRD.2003.04.0043

Lavela, S. L., Weaver, F. M., Smith, B., & Chen, K. (2006). Disease prevalence and use of preventive services: comparison of female veterans in general and those with spinal cord injuries and disorders. *J Womens Health (Larchmt), 15*(3), 301-311. doi:10.1089/jwh.2006.15.301

Le Liepvre, H., Dinh, A., Idiard-Chamois, B., Chartier-Kastler, E., Phé, V., Even, A., . . . Denys, P. (2017). Pregnancy in spinal cord-injured women, a cohort study of 37 pregnancies in 25 women. *Spinal Cord, 55*(2), 167-171. doi:10.1038/sc.2016.138

Lew, S. M., & Kothbauer, K. F. (2007). Tethered Cord Syndrome: An Updated Review. *Pediatric Neurosurgery, 43*(3), 236-248. doi:10.1159/000098836

Lindan, R., Joiner, E., Freehafer, A., & Hazel, C. (1980). Incidence and clinical features of autonomic dysreflexia in patients with spinal cord injury. *Spinal Cord, 18*(5), 285-292.

Linstow, M. v., Biering-Sørensen, I., Liebach, A., Lind, M., Seitzberg, A., Hansen, R. B., & Biering-Sørensen, F. (2014). Spina bifida and sexuality. *Journal of Rehabilitation Medicine, 46*(9), 891-897.

Mauer, U. M., Freude, G., Danz, B., & Kunz, U. (2008). Cardiac-gated phase-contrast magnetic resonance imaging of cerebrospinal fluid flow in the diagnosis of idiopathic syringomyelia. *Neurosurgery, 63*(6), 1139-1144; discussion 1144. doi:10.1227/01.Neu.0000334411.93870.45

Mayer, G. (2017). *S3-Leitlinie Nicht erholsamer Schlaf/Schlafstörungen - Kapitel "Schlafbezogene Atmungsstörungen bei Erwachsenen"*. Retrieved from <https://www.awmf.org/leitlinien/detail/ll/063-001.html>

Mayer, G. (2020). *Insomnie bei neurologischen Erkrankungen, S2k-Leitlinie*. Retrieved from <https://www.dgn.org/leitlinien>

McDermott, A. M., Toelle, T. R., Rowbotham, D. J., Schaefer, C. P., & Dukes, E. M. (2006). The burden of neuropathic pain: results from a cross‐sectional survey. *European Journal of Pain, 10*(2), 127-127. Retrieved from <https://www.sciencedirect.com/science/article/abs/pii/S1090380105000236?via%3Dihub>

McDonnell, G. V., & McCann, J. P. (2000). Issues of medical management in adults with spina bifida. *Childs Nerv Syst, 16*(4), 222-227. doi:10.1007/s003810050502

McKinley, W., Santos, K., Meade, M., & Brooke, K. (2007). Incidence and outcomes of spinal cord injury clinical syndromes. *J Spinal Cord Med, 30*(3), 215-224. doi:10.1080/10790268.2007.11753929

Meier, C., Boes, S., Gemperli, A., Gmünder, H. P., Koligi, K., Metzger, S., . . . Scheel-Sailer, A. (2019). Treatment and cost of pressure injury stage III or IV in four patients with spinal cord injury: the Basel Decubitus Concept. *Spinal Cord Ser Cases, 5*, 30. doi:10.1038/s41394-019-0173-0

Michel, F., Ketter, G., Tiedemann, S., Marcus, O., Hirschfeld, S., Landscheid, M., . . . Raab, A. M. (2022). *Atmung, Atemunterstützung und Beatmung bei akuter und chronischer Querschnittlähmung. Entwicklungsstufe S2e, AWMF-Register Nr. 179-013.* Retrieved from

Miles, A., Jamieson, G., Shasha, L., & Davis, K. (2019). Characterizing dysphagia after spinal surgery. *J Spinal Cord Med*, 1-9. doi:10.1080/10790268.2019.1665613

Monasterio, E. A., Barber, D. B., Rogers, S. J., Able, A. C., & Fredrickson, M. D. (2000). Latex allergy in adults with spinal cord injury: a pilot investigation. *J Spinal Cord Med, 23*(1), 6-9. doi:10.1080/10790268.2000.11753501

Morton, C., Le, J. T., Shahbandar, L., Hammond, C., Murphy, E. A., & Kirschner, K. L. (2013). Pregnancy outcomes of women with physical disabilities: a matched cohort study. *PM R, 5*(2), 90-98. doi:10.1016/j.pmrj.2012.10.011

Nance, D. M., & Sanders, V. M. (2007). Autonomic innervation and regulation of the immune system (1987–2007). *Brain, behavior, and immunity, 21*(6), 736-745.

Nash, M. S., & Bilzon, J. L. J. (2018). Guideline Approaches for Cardioendocrine Disease Surveillance and Treatment Following Spinal Cord Injury. *Current physical medicine and rehabilitation reports, 6*(4), 264-276. doi:10.1007/s40141-018-0203-z

Nash, M. S., & Mendez, A. J. (2007). A guideline-driven assessment of need for cardiovascular disease risk intervention in persons with chronic paraplegia. *Arch Phys Med Rehabil, 88*(6), 751-757. doi:10.1016/j.apmr.2007.02.031

New, P. W. (2016). Secondary conditions in a community sample of people with spinal cord damage. *The Journal of Spinal Cord Medicine, 39*(6), 665-670. doi:10.1080/10790268.2016.1138600

NICE, & National Institute for Health and Care Excellence. (2014). *Pressure ulcers: prevention and management*. Retrieved from United Kingdom:

Nielsen, S. D., Faaborg, P. M., Christensen, P., Krogh, K., & Finnerup, N. B. (2017). Chronic abdominal pain in long-term spinal cord injury: a follow-up study. *Spinal Cord, 55*(3), 290-293. Retrieved from <https://www.nature.com/articles/sc2016124.pdf>

Oleson, C. V., Marino, R. J., Formal, C. S., Modlesky, C. M., & Leiby, B. E. (2020). The effect of zoledronic acid on attenuation of bone loss at the hip and knee following acute traumatic spinal cord injury: a randomized-controlled study. *Spinal Cord*. doi:10.1038/s41393-020-0431-9

Pannek, J. (2011). Treatment of urinary tract infection in persons with spinal cord injury: guidelines, evidence, and clinical practice. A questionnaire-based survey and review of the literature. *J Spinal Cord Med, 34*(1), 11-15. doi:10.1179/107902610x12886261091839

Park, H. J., Noh, S. E., Kim, G. D., & Joo, M. C. (2013). Plain abdominal radiograph as an evaluation method of bowel dysfunction in patients with spinal cord injury. *Annals of Rehabilitation Medicine, 37*(4), 547. Retrieved from <https://www.ncbi.nlm.nih.gov/pmc/articles/PMC3764350/pdf/arm-37-547.pdf>

Pedaballe, A. R., Chhabra, H. S., Tandon, V., Chauhan, P., & Verma, R. (2018). Acute traumatic cervical spinal cord injury in a third-trimester pregnant female with good maternal and fetal outcome: a case report and literature review. *Spinal Cord Series and Cases, 4*, 93-93. doi:10.1038/s41394-018-0127-y

Pellicane, A. J., Wysocki, N. M., & Schnitzer, T. J. (2010). Prevalence of 25-hydroxyvitamin D deficiency in the outpatient rehabilitation population. *American Journal of Physical Medicine & Rehabilitation, 89*(11), 899-904.

Pereira, L. (2003). Obstetric management of the patient with spinal cord injury. *Obstet Gynecol Surv, 58*(10), 678-687. doi:10.1097/01.Ogx.0000086419.94466.76

Petry, K. U., Köchel, H., Bode, U., Schedel, I., Niesert, S., Glaubitz, M., . . . Kühnle, H. (1996). Human papillomavirus is associated with the frequent detection of warty and basaloid high-grade neoplasia of the vulva and cervical neoplasia among immunocompromised women. *Gynecologic oncology, 60*(1), 30-34. Retrieved from <https://www.sciencedirect.com/science/article/abs/pii/S0090825896900074?via%3Dihub>

Prehn-Kristensen, A. (2018). *Nichtorganische Schlafstörungen* Retrieved from <https://www.awmf.org/leitlinien/detail/ll/028-012.html>

Previnaire, J. G., De Bont, N., Bordi, H., Senal, N., & Mortier, P. E. (2018). Open surgery for haemorrhoids in persons with spinal cord injury. *Spinal Cord Series and Cases, 4*, 35-35. doi:10.1038/s41394-018-0070-y

Robertson, K., Dawood, R., & Ashworth, F. (2020). Vaginal delivery is safely achieved in pregnancies complicated by spinal cord injury: a retrospective 25-year observational study of pregnancy outcomes in a national spinal injuries centre. *BMC Pregnancy and Childbirth, 20*(1), 56. doi:10.1186/s12884-020-2752-2

Rotter, K. P., & Larraín, C. G. (2003). Gallstones in spinal cord injury (SCI): a late medical complication? *Spinal Cord, 41*(2), 105-108. doi:10.1038/sj.sc.3101408

Rove, K. O., Husmann, D. A., Wilcox, D. T., Vricella, G. J., & Higuchi, T. T. (2017). Systematic review of bladder cancer outcomes in patients with spina bifida. *J Pediatr Urol, 13*(5), 456.e451-456.e459. doi:10.1016/j.jpurol.2017.05.006

Sauer, P. M., & Harvey, C. J. (1993). Spinal cord injury and pregnancy. *J Perinat Neonatal Nurs, 7*(1), 22-34. doi:10.1097/00005237-199306000-00005

Scheel-Sailer, A., Bartholet, C., Bersch-Porada, I., Curt, A., Gisler, A., Huber, B., . . . Witschi, W. (2018). *Rehabilitation der unteren Exremität, der Steh- und Gehfunktion bei Menschen mit Querschnittlähmung*. Retrieved from

Schnitzer, T. J., Kim, K., Marks, J., Yeasted, R., Simonian, N., & Chen, D. (2016). Zoledronic Acid Treatment After Acute Spinal Cord Injury: Results of a Randomized, Placebo-Controlled Pilot Trial. *PM R, 8*(9), 833-843. doi:10.1016/j.pmrj.2016.01.012

Schurch, B., Wichmann, W., & Rossier, A. B. (1996). Post-traumatic syringomyelia (cystic myelopathy): a prospective study of 449 patients with spinal cord injury. *J Neurol Neurosurg Psychiatry, 60*(1), 61-67. doi:10.1136/jnnp.60.1.61

Shafazand, S., Anderson, K. D., & Nash, M. S. (2019). Sleep complaints and sleep quality in spinal cord injury: a web-based survey. *Journal of Clinical Sleep Medicine, 15*(05), 719-724.

Shapiro, J., Smith, B., Beck, T., Ballard, P., Dapthary, M., BrintzenhofeSzoc, K., & Caminis, J. (2007). Treatment with zoledronic acid ameliorates negative geometric changes in the proximal femur following acute spinal cord injury. *Calcif Tissue Int, 80*(5), 316-322. doi:10.1007/s00223-007-9012-6

Shin, J. C., Chang, S. H., Hwang, S. W., & Lee, J. J. (2018). The Nutritional Status and the Clinical Outcomes of Patients With a Spinal Cord Injury Using Nutritional Screening Tools. *Ann Rehabil Med, 42*(4), 591-600. doi:10.5535/arm.2018.42.4.591

Sigrist-Nix, D., Bersch-Porada, I., Debecker, I., Rupp, R., Schibli, S., Schwager, C., & Tomaschek, R. (2020). *Verbesserung der Funktionsfähigkeit der oberen Extremitäten bei zervikaler Querschnittlähmung. Entwicklungsstufe S2e, AWMF-Register Nr. 179-013.* Retrieved from

Silberstein, M., & Hennessy, O. (1992). Cystic cord lesions and neurological deterioration in spinal cord injury: operative considerations based on magnetic resonance imaging. *Spinal Cord, 30*(9), 661-668. doi:10.1038/sc.1992.130

Simpson, G., Anwar, S., Wilson, J., & Bertapelle, T. (2006). Improving the rehabilitative management of client sexual health concerns after neurological disability: evaluation of a staff sexuality training programme in New Zealand. *Clinical rehabilitation, 20*(10), 847-859. Retrieved from <https://journals.sagepub.com/doi/pdf/10.1177/0269215506072180>

Singh, G., & Triadafilopoulos, G. (2000). Gastroesophageal reflux disease in patients with spinal cord injury. *The Journal of Spinal Cord Medicine, 23*(1), 23-27. doi:10.1080/10790268.2000.11753504

Soleyman-Jahi, S., Yousefian, A., Maheronnaghsh, R., Shokraneh, F., Zadegan, S. A., Soltani, A., . . . Rahimi-Movaghar, V. (2018). Evidence-based prevention and treatment of osteoporosis after spinal cord injury: a systematic review. *European Spine Journal, 27*(8), 1798-1814. Retrieved from <https://link.springer.com/content/pdf/10.1007/s00586-017-5114-7.pdf>

Spina Bifida Association. (2018). Guidelines for the Care of People with Spina Bifida. Retrieved from <https://www.spinabifidaassociation.org/guidelines/>

Spina Bifida Association. (2020). Guidelines for the Care of People with Spina Bifida. Retrieved from <https://www.spinabifidaassociation.org/guidelines/>

Steffen, F., Schulz, B., Wang, H., Gottschalk, S., Grüter, F., Friedrich, J., . . . Weidner, N. (2019). Management of pain in individuals with spinal cord injury: Guideline of the German-Speaking Medical Society for Spinal Cord Injury. *Ger Med Sci, 17*, Doc05. doi:10.3205/000271

Sterling, L., Keunen, J., Wigdor, E., Sermer, M., & Maxwell, C. (2013). Pregnancy outcomes in women with spinal cord lesions. *J Obstet Gynaecol Can, 35*(1), 39-43. doi:10.1016/s1701-2163(15)31046-x

Swaroop, V. T., & Dias, L. S. (2009). Strategies of hip management in myelomeningocele: to do or not to do. *Hip Int, 19 Suppl 6*, S53-55. doi:10.1177/112070000901906s09

Tate, D. G., Wheeler, T., Lane, G. I., Forchheimer, M., Anderson, K. D., Biering-Sorensen, F., . . . Kennelly, M. J. (2020). Recommendations for evaluation of neurogenic bladder and bowel dysfunction after spinal cord injury and/or disease. *The Journal of Spinal Cord Medicine, 43*(2), 141-164. Retrieved from <https://www.tandfonline.com/doi/pdf/10.1080/10790268.2019.1706033?needAccess=true>

Telles, S. C. L., Alves, R. C., & Chadi, G. (2011). Periodic limb movements during sleep and restless legs syndrome in patients with ASIA A spinal cord injury. *Journal of the Neurological Sciences, 303*(1), 119-123. doi:10.1016/j.jns.2010.12.019

Teng, B. J., Song, S. H., Svircev, J. N., Dominitz, J. A., & Burns, S. P. (2018). Colorectal cancer screening in patients with spinal cord injury yields similar results to the general population with an effective bowel preparation: a retrospective chart audit. *Spinal Cord, 56*(3), 226-231. doi:10.1038/s41393-017-0025-3

van Drongelen, S., de Groot, S., Veeger, H. E., Angenot, E. L., Dallmeijer, A. J., Post, M. W., & van der Woude, L. H. (2006). Upper extremity musculoskeletal pain during and after rehabilitation in wheelchair-using persons with a spinal cord injury. *Spinal Cord, 44*(3), 152-159. doi:10.1038/sj.sc.3101826

van Drongelen, S., van der Woude, L. H., Janssen, T. W., Angenot, E. L., Chadwick, E. K., & Veeger, D. H. (2005). Glenohumeral contact forces and muscle forces evaluated in wheelchair-related activities of daily living in able-bodied subjects versus subjects with paraplegia and tetraplegia. *Arch Phys Med Rehabil, 86*(7), 1434-1440. doi:10.1016/j.apmr.2005.03.014

van Gorp, S., Kessels, A. G., Joosten, E. A., van Kleef, M., & Patijn, J. (2015). Pain prevalence and its determinants after spinal cord injury: a systematic review. *Eur J Pain, 19*(1), 5-14. doi:10.1002/ejp.522

van Hylckama Vlieg, A., Helmerhorst, F. M., & Rosendaal, F. R. (2010). The risk of deep venous thrombosis associated with injectable depot-medroxyprogesterone acetate contraceptives or a levonorgestrel intrauterine device. *Arterioscler Thromb Vasc Biol, 30*(11), 2297-2300. doi:10.1161/atvbaha.110.211482

Varacallo, M. D., DD; Pizzutillo, P;. (2023). *Osteoporosis in Spinal Cord Injuries*: StatPearls.

Waites, K. B., Canupp, K. C., Chen, Y. Y., DeVivo, M. J., & Nahm, M. H. (2008). Revaccination of adults with spinal cord injury using the 23-valent pneumococcal polysaccharide vaccine. *J Spinal Cord Med, 31*(1), 53-59. doi:10.1080/10790268.2008.11753981

Weidner, N., Baumberger, M., Göggelmann, C., Marcus, O., Wittgruber, G., & Wildburger, R. (2020). *Thromboembolieprophylaxe bei Querschnittlähmung. Entwicklungsstufe S1.* Retrieved from

White, B. A. B., Dea, N., Street, J. T., Cheng, C. L., Rivers, C. S., Attabib, N., . . . Dvorak, M. F. (2017). The Economic Burden of Urinary Tract Infection and Pressure Ulceration in Acute Traumatic Spinal Cord Injury Admissions: Evidence for Comparative Economics and Decision Analytics from a Matched Case-Control Study. *J Neurotrauma, 34*(20), 2892-2900. doi:10.1089/neu.2016.4934

Wong, S., Derry, F., Grimble, G., & Forbes, A. (2012). How do spinal cord injury centres manage malnutrition? A cross-sectional survey of 12 regional centres in the United Kingdom and Ireland. *Spinal Cord, 50*(2), 132-135. doi:10.1038/sc.2011.118

Wong, S., Derry, F., Jamous, A., Hirani, S. P., Grimble, G., & Forbes, A. (2012). The prevalence of malnutrition in spinal cord injuries patients: a UK multicentre study. *Br J Nutr, 108*(5), 918-923. doi:10.1017/S0007114511006234

World Health Organization. (2015). *WHO statement onf Progestogen-only implants*. Retrieved from

Yagi, M., Hasegawa, A., Takemitsu, M., Yato, Y., Machida, M., & Asazuma, T. (2015). Incidence and the risk factors of spinal deformity in adult patient after spinal cord injury: a single center cohort study. *Eur Spine J, 24*(1), 203-208. doi:10.1007/s00586-014-3534-1

Yang, J., Boninger, M. L., Leath, J. D., Fitzgerald, S. G., Dyson-Hudson, T. A., & Chang, M. W. (2009). Carpal Tunnel Syndrome in Manual Wheelchair Users with Spinal Cord Injury: A Cross-Sectional Multicenter Study. *American Journal of Physical Medicine & Rehabilitation, 88*(12), 1007-1016. doi:10.1097/PHM.0b013e3181bbddc9
